# An aza-Diels–Alder approach to nitrogen-containing tetrabenzoacene derivatives†

**DOI:** 10.1039/d3ra07136g

**Published:** 2024-09-06

**Authors:** Ethan R. Peng, Anthony M. Burke, David J. Dibble, Chandra B. KC, Reina Kurakake, Panyiming Liu, Robert Lopez, Philip R. Dennison, Alon A. Gorodetsky

**Affiliations:** a Department of Materials Science and Engineering, University of California, Irvine Irvine CA 92697 USA alon.gorodetsky@uci.edu; b Department of Chemistry, University of California, Irvine Irvine CA 92697 USA; c Department of Chemical and Biomolecular Engineering, University of California, Irvine Irvine CA 92697 USA

## Abstract

Acenes and *N*-heteroacenes have been synthesized and studied for over a century because of their fundamentally interesting materials properties and promise for device applications. Within this context, our laboratory has previously synthesized nitrogen-containing tetrabenzo[*de*,*hi*,*op*,*st*]pentacenes *via* an aza-Diels–Alder reaction-based approach, and herein, we expand our methodology to obtain substituted, expanded, functionalized, and dimeric tetrabenzoacenes. Overall, our study adds to the limited number of tetrabenzoacene derivatives reported to date and may open further opportunities for these materials in organic optoelectronics applications.

## Abstract 2

We report a general and modular aza-Diels–Alder reaction-based approach to substituted, expanded, functionalized, and dimeric nitrogen-containing tetrabenzoacenes.

## Graphical abstract

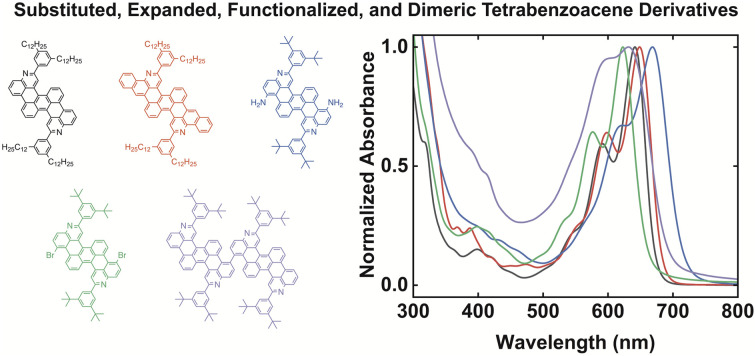

## Introduction

Acenes, which are compounds that consist of linearly fused benzene rings and possess a single aromatic sextet, have been extensively studied for >100 years.^1–19^ These polycyclic aromatic hydrocarbons are widely investigated as organic functional materials because of their fundamentally interesting electronic structures and photophysical properties^1–3,12,15,17^ as well as because of their demonstrated potential for thin film transistor and organic optoelectronics applications.^1,2,9,19^ Several synthetic routes to acene variants such as pentacene have therefore been reported, including nucleophilic addition or direct reduction of acene quinones, oxidation/dehydrogenation of hydroacenes, and retrocycloaddition of bridged acene precursors.^1,2,4,12,14,17^ Within this context, *N*-heteroacenes, which are a subset of acenes containing nitrogen atoms, have also attracted much attention from researchers.^1,4–8,10–13,16,18^ These nitrogen-containing molecules often exhibit enhanced oxidative stability, additional electronic tunability, ambipolar charge transport in transistors, and improved light absorption/emission in optoelectronic devices (all relative to acenes).^1,5,7,10–12,16–18^ Several synthetic routes to *N*-heteroacene derivatives have accordingly been reported, including the direct oxidation of hydroazaacenes, condensation of diamines with orthoquinones or dihydroxy compounds, and Pd catalyzed coupling of aromatic diamines with activated aryl halides.^4–6,8,11–13,18^ Consequently, there exists significant motivation for the continued development of facile approaches to the synthesis of *N*-heteroacene derivatives.

**Fig. 1:**
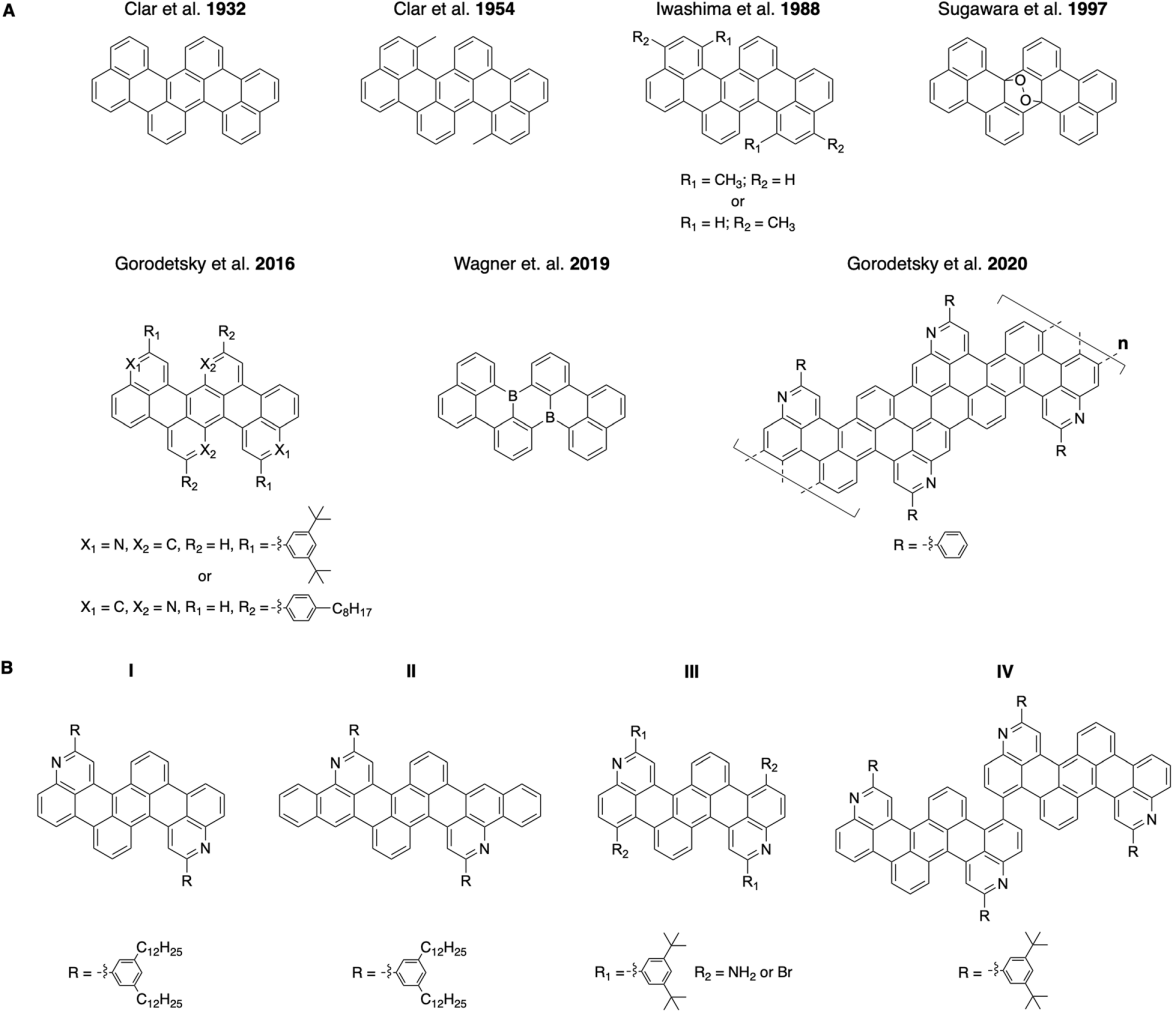
(A) Top row: the previously-reported all-carbon and oxidized tetrabenzo[*de*,*hi*,*op*,*st*]pentacenes. Bottom row: the previously-reported nitrogen- and boron-containing monomeric or polymeric tetrabenzo[*de*,*hi*,*op*,*st*]pentacenes. (B) The tetrabenzoacene derivatives reported in this work.

Correspondingly, tetrabenzo[*de*,*hi*,*op*,*st*]pentacenes, which are acene variants with benzene rings fused to a linear pentacene core, have been investigated intermittently for >90 years (Fig. 1A, top row).^20–31^ These molecules were viewed as interesting because of their surprising photochromic behavior when oxidized, impressive thermal stabilities, and unexpected electrical properties.^24,25,27,30,31^ Such all-carbon tetrabenzopentacenes were typically obtained through base-mediated cyclodehydrohalogenation of chlorinated dinapthylanthracene precursors and could be further converted into their endoperoxide forms by reversible oxidation.^21,25,26,30^ Most recently, monomeric and polymeric heterotetrabenzo[*de*,*hi*,*op*,*st*]pentacenes, which contain nitrogen or boron atoms, have been reported by different laboratories (Fig. 1A, bottom row).^32–34^ These molecules and polymers exhibited favorable photophysical properties, altered electrochemical characteristics, and distinct electronic structures.^32–34^ Such nitrogen- and boron-containing tetrabenzopentacene variants were obtained through base-mediated cyclodehydrohalogenation, nickel-mediated Yamamoto-type dehalogenation, or surface-assisted cyclodehydrogenation of various corresponding anthracene precursors.^32–34^ To date, tetrabenzopentacenes of any kind are accessible through only a few synthetic routes, whose scope and modularity have not been adequately explored.

Herein, we report a general and modular aza-Diels–Alder reaction-based approach to substituted, expanded, functionalized, and dimeric nitrogen-containing tetrabenzoacenes (Fig. 1B). First, we synthesize a nitrogen-containing tetrabenzopentacene bearing pendant phenyl rings substituted with dodecyl chains, thus demonstrating incorporation of solubilizing functionalities (Fig. 1B, compound I). Second, we synthesize a nitrogen-containing tetrabenzoheptacene, thus describing expansion of the molecular aromatic core of our compound (Fig. 1B, compound II). Third, we synthesize a nitrogen-containing tetrabenzopentacene featuring amino and bromo functional groups, thus showing installation of reactive electron-withdrawing and electron-donating handles (Fig. 1B, compound III). Fourth, we synthesize a dimeric nitrogen-containing tetrabenzopentacene, thus furnishing a potentially valuable larger model compound (Fig. 1B, compound IV). Last, we comparatively investigate the electronic properties of our various tetrabenzoacene derivatives with ultraviolet-visible (UV-Vis) spectroscopy. Our synthetic strategy affords multiple new nitrogen-containing tetrabenzo[*de*,*hi*,*op*,*st*]pentacene variants and may enhance the utility of this class of molecules in organic optoelectronics applications.

## Results

**Scheme 1:**
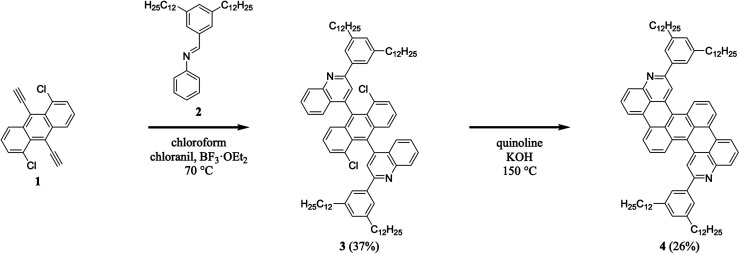
The synthesis of dodecyl-substituted tetrabenzopentacene 4.

We began our efforts by synthesizing a nitrogen-containing tetrabenzopentacene derivative bearing pendant phenyl rings substituted with dodecyl chains (Scheme 1). First, we reacted 1,5-dichloro-9,10-diethynylanthracene 1 with 3,5-didodecylphenyl-substituted phenylaldimine 2 under our highly optimized general aza-Diels–Alder reaction conditions (*i.e.*, chloroform as the solvent, BF_3_·OEt_2_ as the Lewis acid catalyst, and chloranil as the oxidant), forming two quinoline moieties and obtaining intermediate 3 in a reasonable yield of 37% (see Experimental I, II, and ESI Fig. S1–S4†).^32,34–41^ Next, we cyclodehydrohalogenated diquinolineanthracene variant 3 under standard conditions (*i.e.*, quinoline as the solvent and KOH as the base), forming two intramolecular carbon–carbon bonds and furnishing dodecyl-substituted tetrabenzopentacene product 4 in a more modest yield of 26% (see Experimental I, II, and ESI Fig. S5 and S6†).^32,42^ These experiments demonstrated that our tetrabenzopentacene was readily modified with extended alkyl chains, which can mitigate aggregation and enhance solubility for acenes.^1,2,39^

**Scheme 2:**
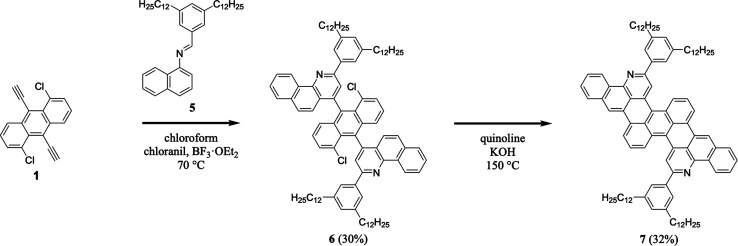
The synthesis of tetrabenzoheptacene 7.

We continued our efforts by synthesizing a nitrogen-containing tetrabenzoheptacene derivative (Scheme 2). First, we reacted 1,5-dichloro-9,10-diethynylanthracene 1 with 3,5-didodecylphenyl-substituted naphthylaldimine 5 under our established general aza-Diels–Alder reaction conditions, forming two benzoquinoline moieties and obtaining intermediate 6 in a reasonable yield of 30% (see Experimental I, II, and ESI Fig. S7–S12†).^32,34–41^ Next, we cyclodehydrohalogenated diquinolineanthracene variant 6 under our validated standard conditions, forming two intramolecular carbon–carbon bonds and furnishing dodecyl-substituted tetrabenzoheptacene product 7 in a comparable yield of 32% (see Experimental I, II, and ESI Fig. S13 and S14†).^32,42^ These experiments demonstrated that our tetrabenzopentacene's core aromatic motif was readily expanded to a heptacene, which can possess improved functional properties and serve as a model longer acene.^3,17,43^

**Scheme 3:**
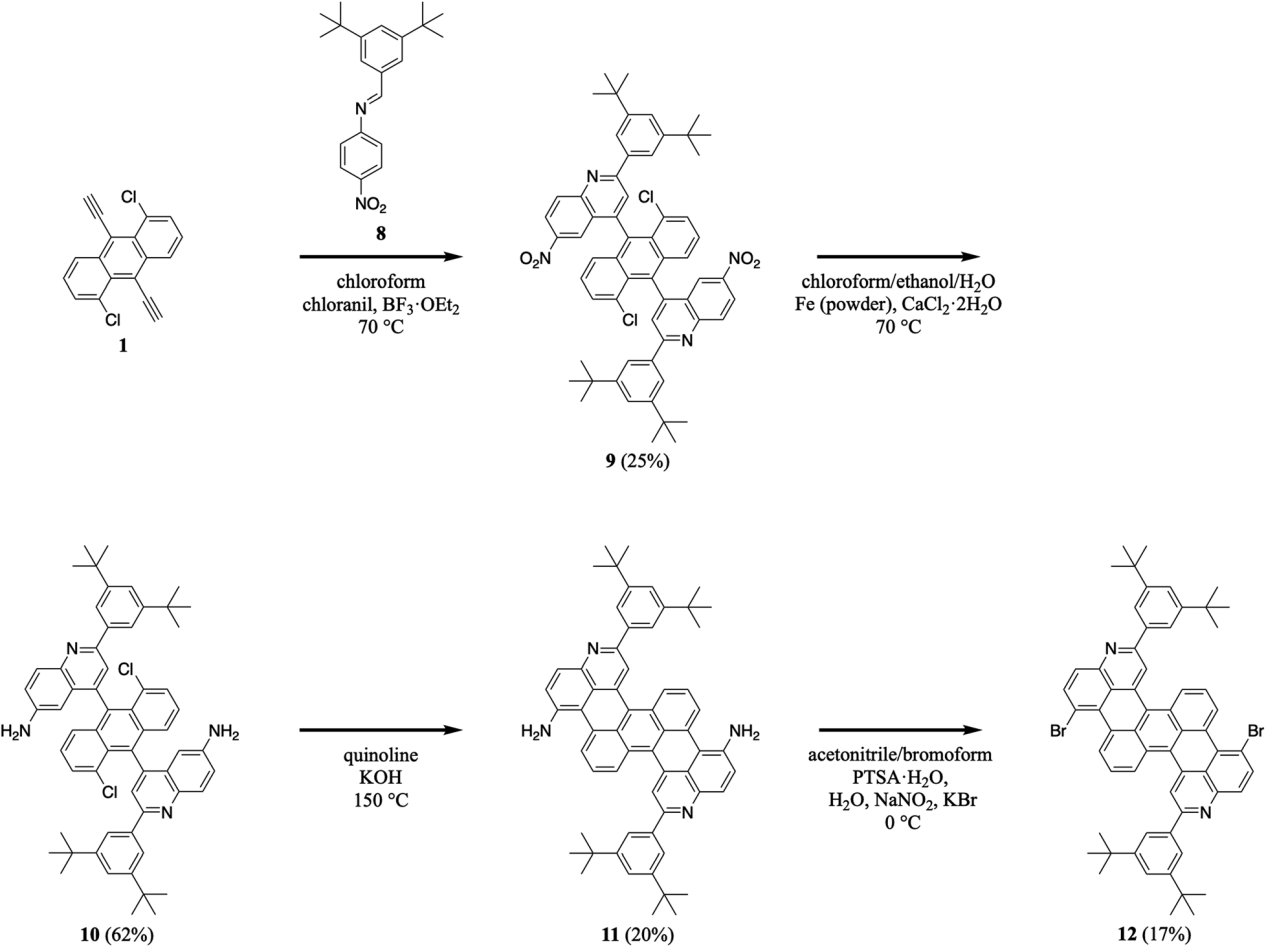
The synthesis of amino-functionalized tetrabenzopentacene 11 and bromo-functionalized tetrabenzopentacene 12.

We extended our efforts by synthesizing nitrogen-containing tetrabenzopentacene derivatives featuring amino and bromo functional groups (Scheme 3). First, we reacted 1,5-dichloro-9,10-diethynylanthracene 1 with 3,5-di-*tert*-butylphenyl-substituted 4-nitrophenylaldimine 8 under our standard aza-Diels–Alder reaction conditions, forming two nitroquinoline moieties and obtaining intermediate 9 in a moderate yield of 25% (see Experimental I, II, and ESI Fig. S15–S20†).^32,34–41^ Next, we reduced 9's nitro groups using routine catalytic transfer hydrogenation conditions (*i.e.*, chloroform/ethanol/water as the solvents and Fe/CaCl_2_ as the reducing reagents), forming two 6-aminoquinoline moieties and generating intermediate 10 in a good yield of 62% (see Experimental I, II, and ESI Fig. S21, and S22†).^44^ Then, we cyclodehydrohalogenated diquinolineanthracene variant 10 under our validated standard conditions, forming two intramolecular carbon–carbon bonds and furnishing amino-functionalized tetrabenzopentacene product 11 in a moderate yield of 20% (see Experimental I, II, and ESI Fig. S23 and S24†).^32,42^ Last, we converted tetrabenzopentacene 11's amino groups to bromo groups using established Sandmeyer-type reaction conditions (*i.e.*, acetonitrile/bromoform as the solvents and NaNO_2_/KBr/PTSA as the acid system), affording bromo-functionalized tetrabenzopentacene product 12 in a moderate yield of 17% (see Experimental I, II, and ESI Fig. S25 and S26†).^45^ These experiments demonstrated that our tetrabenzopentacene was readily substituted with ubiquitous electron-donating and electron-withdrawing groups, which readily tune molecular electronic properties^10,15,16,18^ and can enable various metal-catalyzed cross-coupling or functionalization reactions.^46–49^

**Scheme 4:**
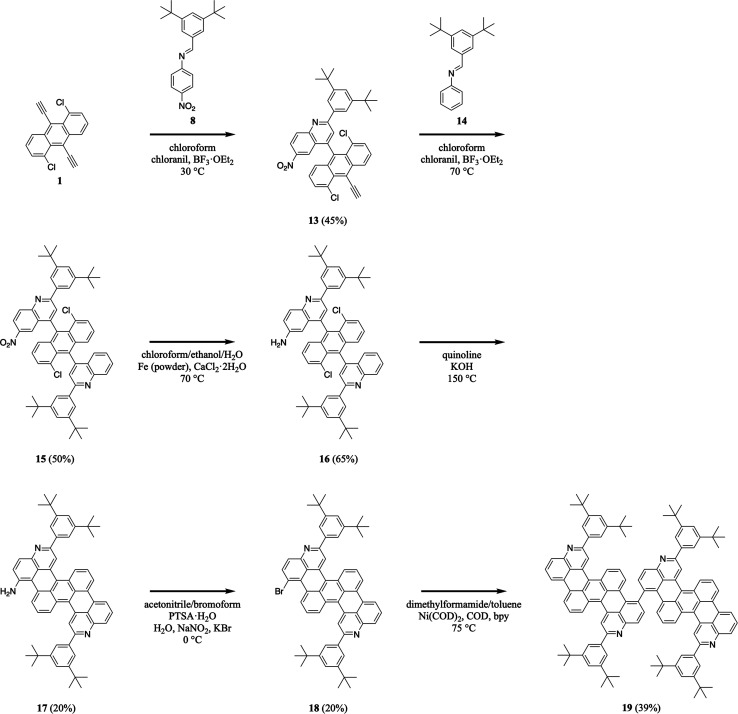
The synthesis of dimeric tetrabenzopentacene 19.

We further advanced our efforts by synthesizing a dimeric nitrogen-containing tetrabenzopentacene derivative (Scheme 4). First, we reacted 1,5-dichloro-9,10-diethynylanthracene 1 with 3,5-di-*tert*-butylphenyl-substituted 4-nitrophenylaldimine 8 under slightly modified milder aza-Diels–Alder reaction conditions (*i.e.*, at a lower temperature), forming a nitroquinoline moiety and generating intermediate 13 in a good yield of 45% (see Experimental I, II, and ESI Fig. S27 and S28†).^32,34–41^ Next, we reacted quinolineanthracene variant 13 with 3,5-di-*tert*-butylphenyl-phenylaldimine 14 under our standard aza-Diels–Alder reaction conditions, forming a quinoline moiety and generating intermediate nitro-functionalized quinolineanthracene variant 15 in a good yield of 50% (see Experimental I, II, and ESI Fig. S29 and S30†).^32,34–41^ Then, we reduced 15's nitro group using routine catalytic transfer hydrogenation conditions, forming amino-functionalized quinolineanthracene variant 16 in a good yield of 65% (see Experimental I, II, and ESI Fig. S31 and S32†).^44^ In turn, we cyclodehydrohalogenated diquinolineanthracene variant 16 under our validated standard conditions, forming two intramolecular carbon–carbon bonds and furnishing intermediate 17 in a moderate yield of 20% (see Experimental I, II, and ESI Fig. S33 and S34†).^32,42^ Subsequently, we converted tetrabenzopentacene 17's amino group to a bromo group using established Sandmeyer-type reaction conditions, affording intermediate 18 in a moderate yield of 20% (see Experimental I, II, and ESI Fig. S35 and S36†).^45^ Last, we cross-coupled the bromo groups of two tetrabenzopentacene 18's with each other using well-known Ullmann reaction conditions (*i.e.*, dimethylformamide/toluene as the solvents and Ni(COD)_2_/COD/bpy as the transition metal catalyst system), affording dimeric tetrabenzopentacene product 19 in a reasonable yield of 39% (see Experimental I, II, and ESI Fig. S37 and S38†).^50^ These experiments demonstrated that our tetrabenzopentacene could be site-specifically substituted with single halogen functional groups, which are amenable to many routine cross-coupling reactions,^46–49^ and moreover could be readily assembled into larger molecular frameworks, which opens opportunities for the synthesis of corresponding nitrogen-containing graphene nanoribbons.^16,34,51^

**Fig. 2:**
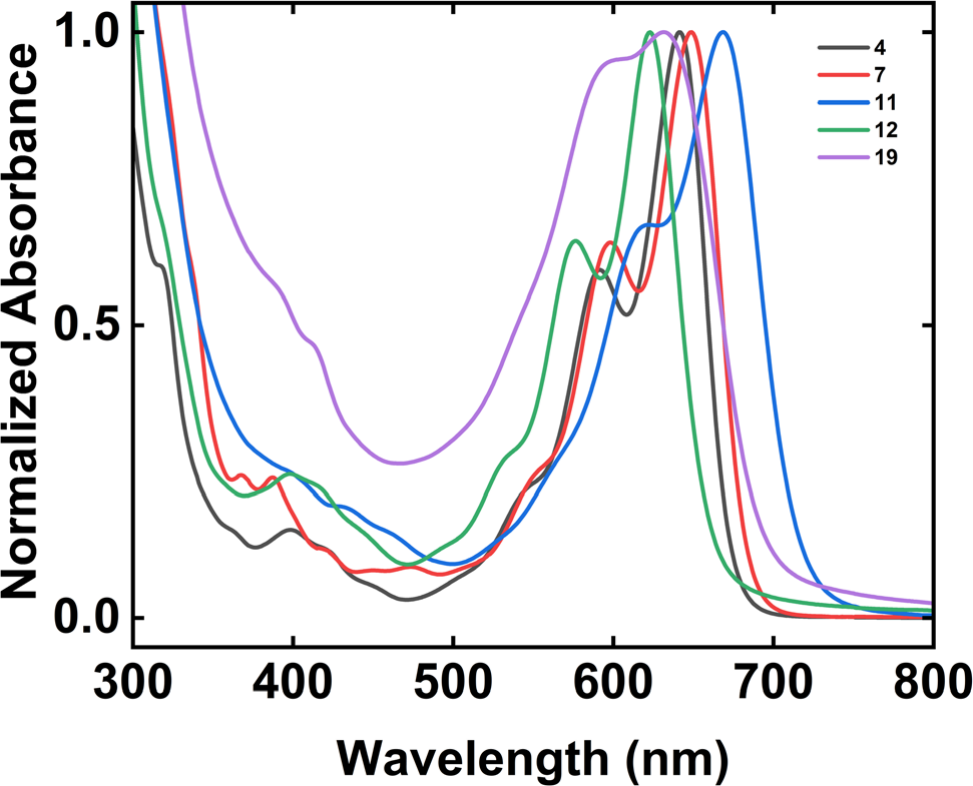
The UV-Vis absorption spectra obtained for 4 (black trace), 7 (red trace), 11 (blue trace), 12 (green trace), and 19 (purple trace). Note that the absorption spectra were normalized by using their maximum absorbance peak within the 600 nm to 700 nm wavelength range in order to facilitate direct comparisons.

We last investigated the electronic properties of our tetrabenzoacene derivatives by means of UV-Vis spectroscopy (Fig. 2). First, the spectrum obtained for dodecyl-substituted tetrabenzopentacene 4 featured prominent absorption peaks with maxima at 641 nm and 592 nm as well as a shoulder at 541 nm, with the addition of extended side chains mitigating aggregation but not significantly shifting the absorption peaks relative to those of the analogous previously-reported *tert*-butyl-substituted tetrabenzopentacene (Fig. 2 and ESI Fig. S39†).^32^ Second, the spectrum obtained for dodecyl-substituted tetrabenzoheptacene 7 featured prominent absorption peaks with maxima at 648 nm and 598 nm as well as a shoulder at 547 nm, with the extension of the aromatic core only slightly red shifting the absorption peaks relative to those of the analogous *tert*-butyl- and dodecyl-substituted tetrabenzopentacenes (Fig. 2 and ESI Fig. S39†). Third, the spectrum obtained for amino-functionalized tetrabenzopentacene 11 featured prominent absorption peaks at 668 nm and 622 nm as well as a small shoulder at 554 nm, with the introduction of the two electron-donating amino groups substantially red-shifting the absorption peaks relative to those of the analogous *tert*-butyl- and dodecyl-substituted tetrabenzopentacenes (Fig. 2 and ESI Fig. S39†). Fourth, the spectrum obtained for bromo-functionalized tetrabenzopentacene 12 featured prominent absorption peaks at 623 nm and 576 nm as well as a shoulder at 521 nm, with the introduction of the two electron-withdrawing bromo groups substantially blue-shifting the absorption peaks relative to those of the analogous *tert*-butyl- and dodecyl-substituted tetrabenzopentacenes (Fig. 2 and ESI Fig. S39†). Last, the spectrum obtained for dimeric tetrabenzopentacene 19 featured prominent absorption peaks at 631 nm and 590 nm and no obvious shoulders, with the covalent linkage of the tetrabenzopentacene moieties causing aggregation and broadening/blue-shifting the absorption peaks relative to those of the monomeric tetrabenzopentacenes in agreement with precedent for some previously-reported acenes (Fig. 2 and ESI Fig. S39†).^52,53^ Overall, these measurements showed that the electronic properties of our nitrogen-containing tetrabenzoacene derivatives could be controlled to some extent *via* the described synthetic strategies.

## Conclusion

In summary, we have described the synthesis and characterization of substituted, expanded, functionalized, and dimeric nitrogen-containing tetrabenzoacenes, and our findings hold significance for several reasons. First, the modification of our tetrabenzopentacenes with extended alkyl chains not only enhances solubility and mitigates aggregation but could also eventually simplify their processing from common organic solvents within the context of transistor applications.^1,2,7,9,10,19^ Second, the straightforward substitution of our tetrabenzopentacenes with electron-donating amino and electron-withdrawing bromo groups readily affords tuning of their optical and electrochemical properties in a manner advantageous for optoelectronics applications.^1,2,11,16,18,19^ Third, the site-specific substitution of our tetrabenzopentacenes with reactive amine and bromine functional handles immediately opens opportunities for their further modification *via* common chemical reactions, *e.g.*, Stille, Suzuki, Heck, and Ullmann couplings.^46–48^ Last, the expansion and dimerization of our tetrabenzopentacenes suggests that they may prove useful as model compounds for nitrogen-containing acenes and could even serve as precursors for nitrogen-doped graphene nanoribbons.^3,16,17,34,43,51^ Overall, our synthetic methodology substantively adds to the small number of tetrabenzoacene derivatives reported to date and may open further opportunities for these materials in organic optoelectronics applications.

## Experimental

### I. General methods

#### A. Materials and conditions

All chemicals and solvents were purchased from Sigma Aldrich, Acros Organics, Combi-Blocks, or Thermo Fisher Scientific. Toluene and chloroform were routinely dried with 3 Å molecular sieves and stored under argon. The glassware was oven dried at temperatures of 150–200 °C. The reactions were performed under dry argon unless otherwise noted. Additional protocols are noted below for specific compounds where appropriate.

#### B. Compound purification

Flash chromatography was performed using a CombiFlash Rf 200 purification system (Teledyne ISCO, Inc.) according to the manufacturer's recommended protocols. Chromatography solvents are reported as percentages followed by solvent combinations. Additional purification-relevant information is noted below for specific compounds where appropriate.

#### C. Nuclear magnetic resonance (NMR) spectroscopy characterization

The intermediates and products were characterized with ^1^H and ^13^C nuclear magnetic resonance (NMR) spectroscopy in the University of California, Irvine Nuclear Magnetic Resonance Facility. The NMR measurements were performed on either a Bruker DRX500 instrument outfitted with a CryoProbe (Bruker TCI 500 MHz, 5 mm diameter tubes) or an AVANCE600 instrument outfitted with a CryoProbe (Bruker CBBFO 600 MHz, 5 mm diameter tubes). The NMR experiments were typically performed at compound concentrations of ∼1 mg mL^−1^ to ∼10 mg mL^−1^. The chemical shifts were reported in ppm for both the ^1^H and ^13^C NMR spectra. The chemical shifts for the NMR data were referenced as follows: for compounds in CDCl_3_, the ^1^H NMR spectra were referenced to tetramethylsilane (TMS) at 0.00 ppm or the residual CHCl_3_ peak at 7.26 ppm, and the ^13^C NMR spectra were referenced to the residual CHCl_3_ peak at 77.16 ppm; for compounds in CS_2_, the ^1^H NMR spectra were referenced to TMS at 0.00 ppm, and the ^13^C NMR spectra were referenced to TMS at 0.00 ppm. Both the ^1^H and ^13^C NMR data were labeled with the chemical shifts, multiplicities (s = singlet, d = doublet, t = triplet, q = quartet, quint = quintet, m = multiplet, br s = broad singlet), coupling constants in Hertz, and integration values. The NMR spectra were processed and analyzed by using the MestReNova software package.

#### D. Mass (MS) spectroscopy characterization

The intermediates and products were characterized with electrospray ionization (ESI) high resolution mass spectrometry (HRMS) or matrix-assisted laser desorption/ionization-time of flight (MALDI-TOF) mass spectrometry at the University of California, Irvine Mass Spectrometry Facility. The HRMS measurements were performed on a Waters LCT Premier time-of-flight instrument, and the MALDI-TOF measurements were performed on an AB SCIEX TOF/TOF™ 5800 series mass spectrometer using a 349 nm Nd:YAG laser with either TCNQ or dithranol as the matrix. The mass spectra were processed and analyzed by using the standard MassLynx and TOF/TOF Series Explorer software packages.

#### E. Ultraviolet-visible (UV-Vis) spectroscopy characterization

The products were characterized with UV-Vis spectroscopy at the University of California, Irvine Laser Spectroscopy Laboratory. The measurements were performed on a Jasco-V670 Absorption Spectrometer. The measurements were conducted in ambient atmosphere at room temperature. The UV-Vis spectroscopy experiments were repeated for at least three independently prepared solutions in CHCl_3_ for each compound at concentrations between ∼10 μM and ∼41 μM (Fig. 2) or at concentrations between ∼35 μM and ∼84 μM (Fig. S39†). The spectra were processed and analyzed with the Jasco Spectra Manager Suite and Origin software packages.

### II. Detailed synthetic protocols

#### A. 1,5-Dichloro-9,10-diethynylanthracene (1)

((1,5-Dichloroanthracene-9,10-diyl)bis(ethyne-2,1-diyl))bis(triisopropylsilane) (0.9770 g, 1.607 mmol) was dissolved in tetrahydrofuran (77 mL) under argon and cooled to −78 °C. Tetrabutylammonium fluoride (1 M, 4.8 mmol) in tetrahydrofuran (4.8 mL) was added dropwise to the solution over 13 minutes, and the mixture was stirred at −78 °C for 1 hour. The mixture was warmed to 0 °C and placed into a dry ice/acetone bath when a color change was observed. The mixture was diluted with a saturated aqueous sodium bicarbonate solution (25 mL), subsequently warmed to room temperature, and further diluted with a saturated sodium bicarbonate solution (50 mL). The reaction mixture was poured into dichloromethane (700 mL), the aqueous and organic phases were separated, and the aqueous phase was extracted with dichloromethane (100 mL). The combined organics were washed with a saturated sodium bicarbonate solution (50 mL) and brine (100 mL). The organics were filtered through a cotton plug, and the solvent was removed *in vacuo*. The crude product was purified by washing with cold hexanes to obtain the desired product (0.4440 g, 94%), and the characterization data matched those previously reported.^32^

#### B. (*E*)-1-(3,5-Didodecylphenyl)-*N*-phenylmethanimine (2)

Aniline (0.21 mL, 2.32 mmol), 3,5-didodecylbenzaldehyde (1.00 g, 2.26 mmol), glacial acetic acid (0.10 mL, 1.75 mmol), and 3 Å molecular sieves (1.00 g) were combined in toluene (30 mL). The mixture was sealed in a pressure vessel and heated at 150 °C for 16 hours. The reaction mixture was cooled and filtered through Celite, and the solvent was removed *in vacuo*. The crude product was isolated without further purification in the yields previously reported (1.10 g, 94%), and the characterization data matched those previously reported.^39^

#### C. 4,4′-(1,5-Dichloroanthracene-9,10-diyl)bis(2-(3,5-didodecylphenyl)quinoline) (3)

Compound 2 (6.57 g, 12.7 mmol), 1,5-dichloro-9,10-diethynylanthracene 1 (1.25 g, 4.23 mmol), chloranil (2.29 g, 9.31 mmol), and BF_3_·OEt_2_ (1.57 mL, 12.7 mmol) were dissolved in chloroform (200 mL) under argon. The reaction mixture was heated at 70 °C for 48 hours. The reaction mixture was cooled, poured into chloroform (300 mL), and washed with a saturated aqueous sodium bicarbonate solution (500 mL × 4) and water (500 mL × 3). The organics were filtered through a cotton plug, and the solvent was removed *in vacuo*. The crude product was triturated with ethanol and purified by flash chromatography (100/0 to 70/30 hexanes/ethyl acetate) to obtain the desired product as two atropisomers (2.05 g (*isomer* 1 + *isomer* 2), 37%). (*isomer* 1): ^1^H NMR (500 MHz, CDCl_3_) *δ* 8.36 (d, *J* = 8.4 Hz, 2H), 8.02 (s, 2H), 7.90 (s, 4H), 7.75 (ddd, *J* = 8.5, 6.8, 1.4 Hz, 2H), 7.51–7.48 (m, 2H), 7.47 (s, 2H), 7.36 (t, *J* = 7.5 Hz, 2H), 7.29–7.27 (m, 2H), 7.13 (t, *J* = 8.1 Hz, 4H), 2.70 (t, *J* = 7.9 Hz, 8H), 1.69 (quint, *J* = 7.7 Hz, 8H), 1.42–1.20 (m, 55H), 0.87 (t, *J* = 6.9 Hz, 12H); (*isomer* 2): ^1^H NMR (500 MHz, CDCl_3_) *δ* 8.36 (d, *J* = 8.5 Hz, 2H), 7.94 (s, 2H), 7.86 (s, 4H), 7.77 (ddd, *J* = 8.3, 6.5, 1.6 Hz, 2H), 7.47 (t, *J* = 7.8 Hz, 4H), 7.43–7.35 (m, 4H), 7.16–7.10 (m, 4H), 2.69 (t, *J* = 7.8 Hz, 8H), 1.68 (quint, *J* = 7.8 Hz, 8H), 1.46–1.07 (m, 76H), 0.87 (t, *J* = 6.9 Hz, 13H); (*isomer* 1): ^13^C NMR (151 MHz, CDCl_3_) *δ* 157.1, 148.6, 148.1, 143.8, 139.5, 134.1, 133.4, 131.2, 130.4, 130.3, 130.1, 129.8, 129.1, 127.8, 127.4, 126.7, 126.3, 125.9, 125.3, 122.5, 36.3, 32.1, 31.9, 29.84, 29.80, 29.72, 29.69, 29.5, 22.8, 14.3; (*isomer* 2): ^13^C NMR (126 MHz, CDCl_3_) *δ* 157.2, 148.7, 148.2, 143.7, 139.5, 134.1, 133.5, 131.3, 130.4, 130.3, 130.1, 129.8, 129.2, 127.7, 127.5, 126.8, 126.5, 125.9, 125.3, 122.3, 36.3, 32.1, 31.9, 29.84, 29.80, 29.71, 29.67, 29.51, 22.8, 14.3; MALDI (dithranol) *m*/*z* calcd for C_92_H_123_Cl_2_N_2_ [M + H]^+^ 1325.9, found 1325.9.

#### D. 2,11-Bis(3,5-didodecylphenyl)dibenzo[*hi*:*st*]dipyrido[2,3,4-*de*:2,3,4-*op*]pentacene (4)

Anhydrous quinoline (38 mL) was added to compound 3 (0.500 g, 0.377 mmol) and powdered KOH (2.54 g, 45.3 mmol) under argon, and the solution was sparged with argon for 15 minutes. The mixture was heated at 150 °C for 2 hours. The reaction mixture was cooled, poured into chloroform (100 mL), and washed with water (100 mL), an aqueous 2.4 M HCl solution (100 mL × 4), water (100 mL × 2), and a saturated aqueous sodium bicarbonate solution (100 mL × 3). The organics were filtered through a cotton plug, and the solvent was removed *in vacuo*. The crude product was purified by flash chromatography (100/0 to 90/10 hexanes/ethyl acetate) to obtain the desired product as a dark blue solid (0.124 g, 26%). ^1^H NMR (600 MHz, CDCl_3_) *δ* 8.58 (d, *J* = 8.6 Hz, 2H), 8.40 (s, 2H), 8.29–8.23 (m, 4H), 8.12 (d, *J* = 8.2 Hz, 2H), 7.82–7.74 (m, 6H), 7.53 (t, *J* = 8.0 Hz, 2H), 7.14 (s, 2H), 2.72 (t, *J* = 7.9 Hz, 8H), 1.71 (quint, *J* = 7.6 Hz, 8H), 1.52–1.00 (m, 72H), 0.86 (t, *J* = 7.0 Hz, 12H); ^13^C NMR (151 MHz, CDCl_3_) *δ* 158.6, 149.6, 143.7, 139.8, 138.6, 130.8, 130.03, 130.00, 129.9, 129.5, 129.23, 129.20, 127.9, 127.7, 126.3, 125.1, 123.7, 121.4, 120.3, 119.4, 36.3, 32.1, 31.7, 29.9, 29.8, 29.7, 29.6, 29.5, 22.8, 14.3; HRMS (ESI) *m*/*z* calcd for C_92_H_121_N_2_ [M + H]^+^ 1253.9530, found 1253.9557.

#### E. *N*-(3,5-Didodecylbenzylidene)naphthalen-1-amine (5)

1-Naphthylamine (2.13 g, 14.9 mmol), 3,5-didodecylbenzaldehyde (6.00 g, 13.6 mmol), and glacial acetic acid (0.10 mL, 1.75 mmol) were dissolved in toluene (200 mL). The vessel containing the solution was equipped with a Dean–Stark trap, and the mixture was refluxed for 24 hours. 3 Å molecular sieves (10.0 g) were added to the solution, and the mixture was refluxed for another 24 hours. The reaction mixture was cooled and filtered through Celite, and the solvent was removed *in vacuo*. The crude product was purified by rinsing with methanol, dissolution in a minimum amount of chloroform, and precipitation from methanol to obtain the desired product (6.13 g, 80%). ^1^H NMR (500 MHz, CDCl_3_) *δ* 8.52 (s, 1H), 8.41–8.36 (m, 1H), 7.90–7.85 (m, 1H), 7.73 (d, *J* = 8.2 Hz, 1H), 7.68 (s, 2H), 7.57–7.51 (m, 2H), 7.48 (t, *J* = 7.8 Hz, 1H), 7.19 (s, 1H), 7.06 (d, *J* = 7.2 Hz, 1H), 2.71 (t, *J* = 7.9 Hz, 4H), 1.71 (quint, *J* = 7.5 Hz, 4H), 1.44–1.23 (m, 37H), 0.92 (t, *J* = 7.0 Hz, 6H); ^13^C NMR (126 MHz, CDCl_3_) *δ* 161.3, 149.8, 143.7, 136.5, 134.0, 132.2, 128.9, 127.7, 126.6, 126.5, 126.2, 125.8, 125.7, 124.2, 112.9, 36.0, 32.1, 31.7, 29.85, 29.81, 29.78, 29.69, 29.55, 29.52, 22.9, 14.3; HRMS (ESI) *m*/*z* calcd for C_41_H_61_NNa [M + Na]^+^ 590.4702, found 590.4702.

#### F. 4,4′-(1,5-Dichloroanthracene-9,10-diyl)bis(2-(3,5-didodecylphenyl)benzo[*h*]quinoline) (6)

Compound 5 (2.71 g, 4.77 mmol), 1,5-dichloro-9,10-diethynylanthracene 1 (0.47 g, 1.59 mmol), chloranil (0.78 g, 3.17 mmol), and BF_3_·OEt_2_ (0.59 mL, 4.78 mmol) were dissolved in chloroform (80 mL) under argon. The reaction mixture was heated at 70 °C for 48 hours. The reaction mixture was cooled, poured into chloroform (100 mL), and washed with a saturated aqueous sodium bicarbonate solution (100 mL × 3) and water (100 mL × 3). The organics were filtered through a cotton plug, and the solvent was removed *in vacuo*. The crude product was triturated with hot ethanol, purified by flash chromatography (100/0 to 80/20 hexanes/chloroform), redissolved in chloroform, and precipitated from ethanol to obtain the desired product as two atropisomers (0.69 g (*isomer* 1 + *isomer* 2), 30%). (*isomer* 1): ^1^H NMR (500 MHz, CDCl_3_) *δ* 9.71 (d, *J* = 8.2 Hz, 2H), 8.16 (s, 2H), 8.07 (s, 4H), 7.90 (d, *J* = 7.9 Hz, 2H), 7.86 (t, *J* = 8.3 Hz, 2H), 7.77–7.73 (m, 2H), 7.64 (d, *J* = 9.1 Hz, 2H), 7.51–7.46 (m, 4H), 7.20 (d, *J* = 8.9 Hz, 2H), 7.16 (s, 2H), 7.12 (dd, *J* = 9.0, 7.2 Hz, 2H), 2.75 (t, *J* = 7.8 Hz, 8H), 1.74 (quint, *J* = 7.6 Hz, 8H), 1.50–1.15 (m, 71H), 0.87 (t, *J* = 7.0 Hz, 12H); (*isomer* 2): ^1^H NMR (500 MHz, CDCl_3_) *δ* 9.71 (d, *J* = 8.2 Hz, 2H), 8.06 (s, 2H), 8.03 (s, 4H), 7.93 (d, *J* = 8.1 Hz, 2H), 7.90–7.84 (m, 2H), 7.79–7.74 (m, 2H), 7.69 (d, *J* = 8.9f Hz, 2H), 7.51–7.45 (m, 4H), 7.30 (d, *J* = 9.0 Hz, 2H), 7.15–7.08 (m, 4H), 2.73 (t, *J* = 7.8 Hz, 8H), 1.72 (quint, *J* = 7.6 Hz, 8H), 1.45–1.22 (m, 74H), 0.87 (t, *J* = 7.0 Hz, 12H); (*isomer* 1): ^13^C NMR (126 MHz, CDCl_3_) *δ* 155.3, 148.5, 146.1, 143.7, 139.6, 134.5, 134.0, 133.5, 132.3, 131.4, 130.4, 130.1, 128.5, 128.0, 127.8, 127.4, 127.3, 126.7, 125.9, 125.4, 125.3, 123.6, 122.4, 36.4, 32.1, 32.0, 29.9, 29.84, 29.82, 29.76, 29.70, 29.5, 22.8, 14.3; (*isomer* 2): ^13^C NMR (126 MHz, CDCl_3_) *δ* 155.4, 148.5, 146.1, 143.7, 139.6, 134.4, 134.0, 133.5, 132.3, 131.5, 130.4, 130.0, 128.5, 128.0, 127.9, 127.7, 127.5, 127.3, 126.7, 126.0, 125.4, 125.2, 123.8, 122.2, 36.4, 32.1, 31.9, 29.86, 29.82, 29.81, 29.74, 29.66, 29.5, 22.8, 14.3; MALDI (dithranol) *m*/*z* calcd for C_100_H_127_Cl_2_N_2_ [M + H]^+^ 1425.9, found 1425.6.

#### G. 2,13-Bis(3,5-didodecylphenyl)dibenzo[*jk*:*yz*]dipyrido[2,3,4-*fg*:2,3,4-*uv*]heptacene (7)

Anhydrous quinoline (35 mL) was added to compound 6 (0.500 g, 0.350 mmol) and powdered KOH (2.36 g, 42.1 mmol) under argon, and the solution was sparged with argon for 15 minutes. The mixture was heated at 150 °C for 2 hours. The reaction mixture was cooled, poured into chloroform (100 mL), and washed with water (100 mL), an aqueous 2.4 M HCl solution (100 mL × 4), water (100 mL × 2), and a saturated aqueous sodium bicarbonate solution (100 mL × 3). The organics were filtered through a cotton plug, and the solvent was removed *in vacuo*. The crude product was purified by flash chromatography (100/0 to 80/20 hexanes/chloroform) to obtain the desired product as a dark blue solid (0.151 g, 32%). ^1^H NMR (500 MHz, CDCl_3_) *δ* 9.32 (d, *J* = 7.5 Hz, 2H), 8.47 (d, *J* = 8.6 Hz, 2H), 8.44 (s, 2H), 8.32 (s, 2H), 8.15 (d, *J* = 7.6 Hz, 2H), 7.94–7.86 (m, 6H), 7.71–7.61 (m, 4H), 7.43 (t, *J* = 8.0 Hz, 2H), 7.15 (s, 2H), 2.76 (t, *J* = 7.8 Hz, 8H), 1.75 (quint, *J* = 7.6 Hz, 8H), 1.48–1.17 (m, 77H), 0.85 (t, *J* = 7.0 Hz, 12H); ^13^C NMR (126 MHz, CDCl_3_) *δ* 156.0, 147.0, 143.5, 139.9, 138.3, 133.8, 131.4, 130.0, 129.8, 129.1, 129.0, 128.5, 128.3, 127.9, 127.5, 127.3, 127.1, 126.1, 125.1, 121.4, 121.0, 120.9, 118.8, 36.3, 32.1, 31.8, 29.89, 29.87, 29.83, 29.80, 29.7, 29.5, 22.8, 14.3; MALDI (TCNQ) *m*/*z* calcd for C_100_H_124_N_2_ [M]^+^ 1353.0, found 1352.7; HRMS (ESI) *m*/*z* calcd for C_100_H_125_N_2_ [M + H]^+^ 1353.9843, found 1353.9814.

#### H. (*E*)-1-(3,5-Di-*tert*-butylphenyl)-*N*-(4-nitrophenyl)methanimine (8)

*p*-Nitro aniline (6.95 g, 50.3 mmol), 3,5-ditertbutylbenzaldehyde (10.0 g, 45.8 mmol), glacial acetic acid (0.10 mL, 1.75 mmol), and 3 Å molecular sieves (20.0 g) were combined in toluene (135 mL). The mixture was sealed in a pressure vessel and heated at 130 °C for 16 hours. The reaction mixture was cooled and filtered through Celite, and the solvent was removed *in vacuo*. The crude product was purified by recrystallization from methanol to obtain the desired product (8.52 g, 55%). ^1^H NMR (600 MHz, CDCl_3_) *δ* 8.42 (s, 1H), 8.27 (dt, *J* = 9.5, 2.5, 2H), 7.76 (d, *J* = 1.9 Hz, 2H), 7.63 (t, *J* = 1.8 Hz, 1H), 7.24 (dt, *J* = 9.4, 2.5, 2H), 1.39 (s, 18H). ^13^C NMR (126 MHz, CDCl_3_) *δ* 163.9, 158.6, 151.9, 145.5, 135.0, 127.1, 125.2, 123.9, 121.4, 35.1, 31.5. MALDI *m*/*z* calcd for C_21_H_27_N_2_O_2_ [M + H]^+^ 339.2, found 339.1.

#### I. 4,4′-(1,5-Dichloroanthracene-9,10-diyl)bis(2-(3,5-di-*tert*-butylphenyl)-6-nitroquinoline) (9)

Compound 8 (6.86 g, 20.3 mmol), 1,5-dichloro-9,10-diethynylanthracene 1 (3.00 g, 10.2 mmol), and chloranil (7.46 g, 30.3 mmol) were dissolved in chloroform (500 mL) under argon. BF_3_·OEt_2_ (3.76 mL, 30.5 mmol) was added to the solution, and the mixture was heated at 70 °C for 16 hours. The reaction mixture was cooled and washed with a saturated aqueous sodium bicarbonate solution (100 mL × 3) and water (100 mL × 3). The organics were filtered through a cotton plug, and the solvent was removed *in vacuo*. The crude product was purified by flash chromatography (100/0 to 90/10 hexanes/ethyl acetate) to obtain the desired product (2.46 g (*isomer* 1 + *isomer* 2), 25%). (*isomer* 1): ^1^H NMR (600 MHz, CDCl_3_) *δ* 8.53 (dd, *J* = 9.2, 2.3 Hz, 2H), 8.50 (d, *J* = 9.2 Hz, 2H), 8.21 (s, 2H), 8.18 (d, *J* = 2.2 Hz, 2H), 8.17–8.15 (m, 4H), 7.65 (s, 2H), 7.55 (d, *J* = 7.1 Hz, 2H), 7.42 (d, *J* = 9.2 Hz, 2H), 7.22 (dd, *J* = 9.1, 7.1 Hz, 2H), 1.44 (s, 34H); (*isomer* 2): ^1^H NMR (600 MHz, CDCl_3_) *δ* 8.55 (dd, *J* = 9.2, 2.5 Hz, 2H), 8.50 (d, *J* = 9.2 Hz, 2H), 8.30 (d, *J* = 2.4 Hz, 2H), 8.12 (d, *J* = 1.8 Hz, 4H), 8.08 (s, 2H), 7.63 (t, *J* = 1.8 Hz, 2H), 7.55 (dd, *J* = 7.1, 1.1 Hz, 2H), 7.41 (dd, *J* = 9.2, 1.1 Hz, 2H), 7.22 (dd, *J* = 9.1, 7.1 Hz, 2H), 1.42 (s, 35H); (*isomer* 1): ^13^C NMR (151 MHz, CDCl_3_) *δ* 169.7, 161.1, 151.9, 150.6, 150.5, 145.6, 141.0, 137.8, 133.6, 132.8, 132.2, 131.1, 131.0, 128.1, 127.6, 127.5, 126.7, 125.4, 124.1, 123.4, 123.0, 122.5, 119.1, 35.3, 31.7; (*isomer* 2): ^13^C NMR (151 MHz, CDCl_3_) *δ* 160.7, 151.9, 150.6, 150.4, 145.8, 141.0, 137.8, 133.5, 132.9, 132.1, 131.2, 131.1, 128.1, 127.6, 127.3, 126.8, 125.3, 123.7, 123.6, 123.2, 122.4, 119.0, 35.3, 31.7, HRMS (ESI) *m*/*z* calcd for C_60_H_56_Cl_2_N_4_O_4_Na [M + Na]^+^ 989.3576, found 989.3599.

#### J. 4,4′-(1,5-Dichloroanthracene-9,10-diyl)bis(2-(3,5-di-*tert*-butylphenyl)quinolin-6-amine) (10)

Compound 9 (1.00 g, 1.03 mmol), Fe powder (3.63 g, 65.0 mmol), and CaCl_2_·2H_2_O (3.17 g, 21.6 mmol) were dissolved in ethanol (400 mL), chloroform (250 mL), and water (40 mL). The mixture was refluxed at 70 °C for 16 hours. The reaction mixture was cooled, filtered through Celite, and washed with water (100 mL × 2). The organics were filtered through a cotton plug, and the solvent was removed *in vacuo*. The crude product was purified by flash chromatography (80/20 hexanes/ethyl acetate) to obtain the desired product (0.5793 g, 62%). ^1^H NMR (500 MHz, CDCl_3_) *δ* 8.18 (d, *J* = 8.8 Hz, 2H), 8.06 (s, 4H), 7.88 (s, 2H), 7.61–7.51 (m, 4H), 7.50 (d, *J* = 7.1 Hz, 2H), 7.19 (dd, *J* = 9.0, 2.4 Hz, 2H), 7.15 (dd, *J* = 9.0, 7.2 Hz, 2H), 6.33 (d, *J* = 2.5 Hz, 2H), 3.77 (br s, 4H), 1.43 (s, 39H). ^13^C NMR (126 MHz, CDCl_3_) *δ* 154.2, 151.3, 144.8, 143.3, 139.2, 134.5, 133.4, 131.5, 131.4, 130.5, 130.4, 128.0, 127.3, 125.7, 123.4, 122.7, 122.5, 121.9, 121.7, 106.6, 35.2, 31.7. MALDI *m*/*z* calcd for C_60_H_61_Cl_2_N_4_ [M + H]^+^ 907.4, found 907.3.

#### K. 2,11-Bis(3,5-di-*tert*-butylphenyl)peryleno[1,2,3-*de*:7,8,9-*d*′*e*′]diquinoline-6,15-diamine (11)

Anhydrous quinoline (70 mL) was added to compound 10 (1.00 g, 1.10 mmol) and powdered KOH (5.65 g, 101 mmol) under argon, and the solution was sparged with argon for 20 minutes. The mixture was heated at 150 °C for 2 hours. The reaction mixture was cooled, poured into chloroform (100 mL), and washed with water (50 mL × 2), an aqueous 2.4 M HCl solution (50 mL × 2), a saturated aqueous sodium bicarbonate solution (50 mL × 2), and water (50 mL × 2). The organics were filtered through a cotton plug, and the solvent was removed *in vacuo*. The crude product was purified by flash chromatography (70/30 hexanes/ethyl acetate) to obtain the desired product as a dark blue solid (0.1840 g, 20%). ^1^H NMR (600 MHz, CDCl_3_) *δ* 8.33 (dd, *J* = 12.1, 8.0 Hz, 4H), 8.26 (s, 2H), 7.94 (d, *J* = 1.7 Hz, 4H), 7.88 (d, *J* = 8.9 Hz, 2H), 7.56 (t, *J* = 1.8 Hz, 2H), 7.39 (t, *J* = 8.0 Hz, 2H), 7.14 (d, *J* = 8.9 Hz, 2H), 4.55 (br s, 4H), 1.43 (s, 41H). ^13^C NMR (151 MHz, CDCl_3_) *δ* 155.2, 151.3, 144.4, 143.3, 139.7, 137.1, 130.4, 130.1, 129.8, 128.2, 127.0, 126.6, 126.2, 125.4, 123.5, 123.3, 121.7, 120.8, 118.5, 112.6, 35.2, 31.7. HRMS (ESI) *m*/*z* calcd for C_60_H_59_N_4_ [M + H]^+^ 835.4740, found 835.4731.

#### L. 6,15-Dibromo-2,11-bis(3,5-di-*tert*-butylphenyl)peryleno[1,2,3-*de*:7,8,9-*d*′*e*′]diquinoline (12)

Compound 11 (0.10 g, 0.12 mmol) and *p*-toluenesulfonic acid monohydrate (1.82 g, 9.57 mmol) were dissolved in CH_3_CN (10 mL) and CHBr_3_ (5 mL) at 0 °C, and the solution was stirred under argon for 10 minutes. NaNO_2_ (0.49 g, 7.1 mmol) and KBr (1.14 g, 9.58 mmol) in water (5 mL) were added to the solution, and the mixture was stirred at 0 °C for 10 minutes. The reaction mixture was diluted with chloroform (50 mL) and washed with a saturated aqueous sodium bicarbonate solution (50 mL × 2) and water (50 mL × 2). The organics were filtered through a cotton plug, and the solvent was removed *in vacuo*. The crude product was purified by flash chromatography (90/10 hexane/ethyl acetate) to obtain the desired product as a dark blue film (0.0193 g, 17%). ^1^H NMR (500 MHz, CDCl_3_) *δ* 9.18 (d, *J* = 7.2 Hz, 2H), 8.53 (d, *J* = 8.7 Hz, 2H), 8.26 (s, 2H), 8.04 (d, *J* = 8.9 Hz, 2H), 8.00–7.94 (m, 6H), 7.61 (t, *J* = 1.8 Hz, 2H), 7.47 (t, *J* = 8.1 Hz, 2H), 1.44 (s, 37H). ^13^C NMR (126 MHz, CDCl_3_) *δ* 158.8, 151.6, 148.0, 139.0, 137.9, 137.3, 129.6, 129.5, 129.1, 128.7, 128.1, 126.9, 126.72, 126.66, 126.5, 125.7, 124.2, 122.1, 119.7, 117.8, 35.3, 31.7. MALDI *m*/*z* calcd for C_60_H_55_Br_2_N_2_ [M + H]^+^ 961.3, found 961.2; HRMS (ESI) *m*/*z* calcd for C_60_H_55_Br_2_N_2_ [M + H]^+^ 961.2732, found 961.2715.

#### M. 2-(3,5-Di-*tert*-butylphenyl)-4-(1,5-dichloro-10-ethynylanthracen-9-yl)-6-nitroquinoline (13)

Compound 8 (3.43 g, 10.1 mmol), 1,5-dichloro-9,10-diethynylanthracene 1 (3.00 g, 10.2 mmol), and chloranil (3.73 g, 15.2 mmol) was dissolved in chloroform (350 mL) under argon. BF_3_·OEt_2_ (1.88 mL, 15.2 mmol) was added to the solution, and the mixture was heated at 30 °C for 16 hours. The reaction mixture was cooled and washed with a saturated aqueous sodium bicarbonate solution (100 mL × 3) and water (100 mL × 3). The organics were filtered through a cotton plug, and the solvent was removed *in vacuo*. The crude product was purified by flash chromatography (90/10 hexanes/ethyl acetate) to obtain the desired product (2.8877 g, 45%). ^1^H NMR (500 MHz, CDCl_3_) *δ* 9.13–9.07 (m, 1H), 8.52–8.42 (m, 3H), 8.09–8.05 (m, 2H), 7.94 (s, 1H), 7.66 (dd, *J* = 7.0, 1.4 Hz, 1H), 7.61 (t, *J* = 1.8 Hz, 1H), 7.57 (s, 1H), 7.56 (d, *J* = 1.8 Hz, 1H), 7.20–7.17 (m, 1H), 7.15–7.11 (m, 1H), 4.38 (s, 1H), 1.44–1.30 (m, 18H). ^13^C NMR (126 MHz, CDCl_3_) *δ* 169.7, 160.7, 151.8, 141.0, 136.7, 133.2, 132.2, 132.0, 131.6, 131.2, 130.74, 130.65, 130.0, 128.1, 127.2, 126.8, 126.5, 125.3, 123.7, 123.4, 123.0, 122.4, 96.6, 35.3, 31.6; MALDI *m*/*z* calcd for C_39_H_33_Cl_2_N_2_O_2_ [M + H]^+^ 631.19, found 631.16.

#### N. (*E*)-1-(3,5-Di-*tert*-butylphenyl)-*N*-phenylmethanimine (14)

Aniline (5.01 mL, 55.4 mmol), 3,5-di-*tert*-butylbenzaldehyde (10.0 g, 45.8 mmol), and *p*-toluenesulfonic acid monohydrate (0.44 g, 2.31 mmol) were dissolved in toluene (250 mL). The vessel containing the solution was equipped with a Dean–Stark trap, and the mixture was refluxed for 16 hours. The reaction mixture was cooled and washed with a saturated aqueous sodium bicarbonate solution (250 mL). The organics were dried with anhydrous sodium sulfate, and the solvent was removed *in vacuo*. The crude product was purified by flash chromatography to obtain the pure product in the yields previously reported (12.0 g, 89%), and the characterization data matched those previously reported.^32^

#### O. 2-(3,5-Di-*tert*-butylphenyl)-4-(1,5-dichloro-10-(2-(3,5-di-*tert*-butylphenyl)quinolin-4-yl)anthracen-9-yl)-6-nitroquinoline (15)

Compound 14 (1.40 g, 4.77 mmol), compound 13 (2.00 g, 3.17 mmol), and chloranil (1.16 g, 4.72 mmol) were dissolved in chloroform (150 mL) under argon. BF_3_·OEt_2_ (0.55 mL, 4.46 mmol) was added to the solution, and the mixture was heated at 70 °C for 16 hours. The reaction mixture was cooled and washed with a saturated aqueous sodium bicarbonate solution (100 mL × 3) and water (100 mL × 3). The organics were poured through a cotton plug, and the solvent was removed *in vacuo*. The crude product was purified by flash chromatography (80/20 hexanes/ethyl acetate) to obtain the desired product (1.4584 g, 50%). ^1^H NMR (500 MHz, CDCl_3_) *δ* 8.59–8.48 (m, 2H), 8.41 (d, *J* = 8.5 Hz, 1H), 8.32 (d, *J* = 2.5 Hz, 1H), 8.14 (d, *J* = 1.9 Hz, 2H), 8.13–8.04 (m, 3H), 7.95 (s, 1H), 7.86–7.75 (m, 1H), 7.68–7.60 (m, 1H), 7.60–7.54 (m, 2H), 7.56–7.49 (m, 3H), 7.49–7.41 (m, 2H), 7.37 (d, *J* = 9.0 Hz, 1H), 7.22–7.13 (m, 2H), 1.46 (s, 18H), 1.44 (s, 18H); ^13^C NMR (126 MHz, CDCl_3_) *δ* 160.8, 157.6, 151.83, 151.82, 151.4, 151.1, 150.4, 148.3, 148.1, 145.6, 138.9, 137.8, 135.2, 133.6, 133.5, 132.1, 131.80, 131.77, 130.9, 130.6, 130.3, 129.9, 129.0, 128.3, 128.1, 127.55, 127.51, 127.47, 127.1, 126.9, 126.6, 126.5, 126.2, 125.3, 124.1, 123.8, 123.4, 122.41, 122.38, 122.2, 122.1, 35.31, 35.29, 35.25, 31.71, 31.67; MALDI *m*/*z* calcd for C_60_H_58_Cl_2_N_3_O_2_ [M + H]^+^ 922.39, found 922.33.

#### P. 2-(3,5-Di-*tert*-butylphenyl)-4-(1,5-dichloro-10-(2-(3,5-di-*tert*-butylphenyl)quinolin-4-yl)anthracen-9-yl)quinolin-6-amine (16)

Compound 15 (2.00 g, 2.17 mmol), Fe powder (3.63 g, 65.0 mmol), and CaCl_2_·2H_2_O (3.17 g, 21.6 mmol) were dissolved in ethanol (400 mL), chloroform (250 mL), and water (40 mL). The mixture was refluxed at 70 °C for 16 hours. The reaction mixture was cooled, filtered through Celite, and washed with water (100 mL × 2). The organics were filtered through a cotton plug, and the solvent was removed *in vacuo*. The crude product was purified by flash chromatography (80/20 hexanes/ethyl acetate) to obtain the desired product (1.2538 g, 65%). ^1^H NMR (500 MHz, CDCl_3_) *δ* 8.39 (d, *J* = 8.0 Hz, 1H), 8.19 (d, *J* = 8.7 Hz, 1H), 8.09 (d, *J* = 1.8 Hz, 2H), 8.02 (d, *J* = 1.8 Hz, 2H), 7.99 (s, 1H), 7.86 (s, 1H), 7.77 (ddd, *J* = 8.4, 6.7, 1.5 Hz, 1H), 7.61–7.45 (m, 6H), 7.43–7.36 (m, 1H), 7.33 (d, *J* = 8.3 Hz, 1H), 7.21 (d, *J* = 8.9 Hz, 1H), 7.18–7.09 (m, 2H), 6.36 (d, *J* = 2.5 Hz, 1H), 3.82 (br s, 2H), 1.42 (s, 18H), 1.41 (s, 18H); ^13^C NMR (126 MHz, CDCl_3_) *δ* 157.8, 151.5, 151.3, 148.1, 144.8, 143.5, 138.9, 133.5, 133.4, 131.6, 131.1, 130.5, 130.40, 130.35, 129.7, 129.1, 128.0, 127.7, 127.5, 127.3, 126.6, 126.3, 125.9, 124.1, 123.4, 122.6, 122.4, 122.1, 121.9, 121.7, 119.2, 106.7, 35.3, 35.2, 31.7; MALDI *m*/*z* calcd for C_60_H_60_Cl_2_N_3_ [M + H]^+^ 892.42, found 892.33.

#### Q. 2,11-Bis(3,5-di-*tert*-butylphenyl)peryleno[1,2,3-*de*:7,8,9-*d*′*e*′]diquinolin-6-amine (17)

Anhydrous quinoline (70 mL) was added to compound 16 (1.0 g, 1.1 mmol) and powdered KOH (5.650 g, 100.7 mmol) under argon, and the solution was sparged with argon for 20 minutes. The mixture was heated at 150 °C for 2 hours. The reaction mixture was cooled, poured into chloroform (100 mL), and washed with water (50 mL × 2), an aqueous 2.4 M HCl solution (50 mL × 2), a saturated aqueous sodium bicarbonate solution (50 mL × 2), and water (50 mL × 2). The organics were filtered through a cotton plug, and the solvent was removed *in vacuo*. The crude product was purified by flash chromatography (70/30 hexanes/ethyl acetate) to obtain the desired product (0.1821 g, 20%). ^1^H NMR (500 MHz, CDCl_3_) *δ* 8.58 (d, *J* = 8.7 Hz, 1H), 8.51 (d, *J* = 7.5 Hz, 1H), 8.49–8.43 (m, 2H), 8.31 (s, 1H), 8.19–8.11 (m, 2H), 8.07–8.02 (m, 2H), 8.00 (d, *J* = 2.0 Hz, 2H), 7.95 (d, *J* = 1.8 Hz, 2H), 7.66 (t, *J* = 7.9 Hz, 1H), 7.62–7.54 (m, 3H), 7.49–7.44 (m, 1H), 7.33 (d, *J* = 9.0 Hz, 1H), 4.81 (br s, 2H), 1.46 (s, 17H), 1.42 (s, 17H); ^13^C NMR (126 MHz, CDCl_3_) *δ* 159.1, 155.6, 151.43, 151.36, 150.6, 149.5, 144.6, 143.9, 139.6, 139.5, 139.1, 130.8, 130.6, 130.0, 129.8, 129.6, 129.4, 129.0, 128.8, 128.6, 127.9, 127.6, 127.0, 126.7, 125.6, 125.3, 123.92, 123.87, 123.83, 123.4, 123.1, 122.1, 121.8, 121.6, 121.3, 121.2, 119.9, 119.5, 118.7, 112.5, 35.3, 35.2, 31.72, 31.70; MALDI *m*/*z* calcd for C_60_H_58_N_3_ [M + H]^+^ 820.46, found 820.50.

#### R. 6-Bromo-2,11-bis(3,5-di-*tert*-butylphenyl)peryleno[1,2,3-*de*:7,8,9-*d*′*e*′]diquinoline (18)

Compound 17 (0.20 g, 0.24 mmol) and *p*-toluenesulfonic acid monohydrate (1.82 g, 9.57 mmol) were dissolved in CH_3_CN (15 mL) and CHBr_3_ (5 mL) at 0 °C, and the solution was stirred under argon for 10 minutes. NaNO_2_ (0.49 g, 7.1 mmol) and KBr (1.14 g, 9.58 mmol) in water (5 mL) were added to the solution, and the mixture was stirred at 0 °C for 10 minutes. The reaction mixture was diluted with chloroform (50 mL) and washed with saturated aqueous sodium bicarbonate solution (50 mL × 2) and water (50 mL × 2). The organics were filtered through a cotton plug, and the solvent was removed *in vacuo*. The crude product was purified by flash chromatography (90/10 hexanes/ethyl acetate) to obtain the desired product (0.0424 g, 20%). ^1^H NMR (500 MHz, CDCl_3_) *δ* 9.43 (d, *J* = 7.6 Hz, 1H), 8.76 (d, *J* = 8.7 Hz, 1H), 8.61 (d, *J* = 8.6 Hz, 1H), 8.49 (s, 1H), 8.44 (s, 1H), 8.33 (t, *J* = 7.7 Hz, 2H), 8.20 (d, *J* = 8.3 Hz, 1H), 8.09 (d, *J* = 9.0 Hz, 1H), 8.04 (d, *J* = 1.8 Hz, 2H), 8.01–7.95 (m, 3H), 7.85 (t, *J* = 7.8 Hz, 1H), 7.71 (t, *J* = 8.1 Hz, 2H), 7.65–7.55 (m, 3H), 1.44 (s, 19H), 1.43 (s, 19H); ^13^C NMR (126 MHz, CDCl_3_) *δ* 159.3, 158.9, 151.6, 151.5, 149.6, 148.1, 139.4, 139.0, 138.7, 137.9, 137.5, 130.8, 130.1, 130.0, 129.6, 129.5, 129.39, 129.37, 129.2, 129.0, 128.7, 128.6, 127.9, 127.8, 127.0, 126.9, 126.8, 126.6, 126.2, 125.7, 124.2, 124.1, 123.8, 122.1, 122.0, 121.7, 120.4, 120.2, 119.2, 117.8, 35.28, 35.26, 31.72, 31.69; MALDI *m*/*z* calcd for C_60_H_56_BrN_2_ [M + H]^+^ 883.36, found 883.34.

#### S. 2,2′,11,11′-Tetrakis(3,5-di-*tert*-butylphenyl)-6,6′-biperyleno[1,2,3-*de*:7,8,9-*d*′*e*′]diquinoline (19)

2,2′-Bipyridyl (0.0703 g, 0.450 mmol) and 1,5-cyclooctadiene (0.05 mL, 0.41 mmol) were dissolved in dimethylformamide (5 mL), and the solution was added to bis(cyclooctadiene)nickel (0.12 g, 0.44 mmol) under argon. The mixture was heated at 60 °C for 30 minutes. Compound 18 (0.20 g, 0.23 mmol) in toluene (10 mL) was added to the solution, and the mixture was heated at 75 °C for 48 hours. The reaction mixture was cooled and washed with an aqueous 1.2 M HCl solution (10 mL), a saturated aqueous sodium bicarbonate solution(10 mL × 2), and water (10 mL × 3). The solvent was removed *in vacuo*. The crude product was purified with flash chromatography (90/10 hexanes/ethyl acetate) and size exclusion chromatography using Bio-bead SX-1 resin (with toluene as the mobile phase) to obtain the desired product as a dark blue film (0.0708 g, 39%). ^1^H NMR (600 MHz, CS_2_) *δ* 8.78 (d, *J* = 8.5 Hz, 2H), 8.64 (d, *J* = 8.6 Hz, 2H), 8.56 (s, 2H), 8.45 (s, 2H), 8.39 (d, *J* = 7.6 Hz, 2H), 8.30 (d, *J* = 7.7 Hz, 2H), 8.24 (d, *J* = 7.7 Hz, 2H), 8.05 (d, *J* = 8.3 Hz, 2H), 8.00 (d, *J* = 8.7 Hz, 2H), 7.97 (d, *J* = 1.6 Hz, 4H), 7.90 (d, *J* = 1.7 Hz, 4H), 7.78 (t, *J* = 8.0 Hz, 2H), 7.72 (t, *J* = 7.9 Hz, 2H), 7.54 (d, *J* = 8.6 Hz, 2H), 7.42 (t, *J* = 1.6 Hz, 2H), 7.34 (t, *J* = 1.5 Hz, 2H), 7.29 (t, *J* = 8.1 Hz, 2H), 1.30 (s, 36H), 1.22 (s, 37H); ^13^C NMR (151 MHz, CS_2_) *δ* 157.7, 157.5, 150.3, 150.2, 149.2, 148.5, 139.7, 138.4, 138.3, 137.84, 137.80, 133.4, 130.5, 130.3, 130.1, 129.8, 129.53, 129.50, 129.4, 129.3, 129.0, 128.9, 128.4, 127.6, 127.5, 127.3, 126.3, 126.2, 126.1, 125.3, 123.4, 123.2, 123.1, 121.71, 121.65, 121.2, 120.0, 118.8, 118.4, 34.5, 34.4, 31.34, 31.26; HRMS (ESI) *m*/*z* calcd for C_120_H_111_N_4_ [M + H]^+^ 1607.8809, found 1607.8804.

## Author contributions

Conceptualization, A. A. G.; methodology, A. A. G., D. J. D.; investigation, E. R. P., A. M. B., D. J. D., C. B. C., R. K., P. D. and R. L. (synthesis, NMR characterization, and MS characterization) and E. R. P. and P. L. (UV-Vis characterization); data curation, E. R. P. (NMR, MS, and UV-Vis curation) and A. M. B. (NMR and MS curation); writing—original draft preparation, A. A. G.; writing—review and editing, A. A. G., E. R. P. and A. M. B.; visualization, E. R. P.; supervision, A. A. G.; project administration, A. A. G.; funding acquisition, A. A. G. All authors have received and reviewed the published version of the manuscript.

## Conflicts of interest

Alon A. Gorodetsky, Anthony M. Burke, and David J. Dibble are listed as inventors on patents US10899711B2 and US11945780B2, which describe the synthesis of tetrabenzoacene-type compounds.

## Supplementary Material

RA-014-D3RA07136G-s001
