# Supplementary material for: An aza-Diels–Alder approach to nitrogen-containing tetrabenzoacene derivatives
Source: RSC Adv. 2024 Sep 6;14(39):28475–86. doi: 10.1039/d3ra07136g (PMC11378750; doi:10.1039/d3ra07136g)
Supplement: RA-014-D3RA07136G-s001 [file RA-014-D3RA07136G-s001.pdf]

Supplementary Information (SI) for RSC Advances.
This journal is © The Royal Society of Chemistry 2024

Electronic Supplementary Information for

An aza-Diels–Alder approach to nitrogen-containing tetrabenzoacene derivatives

Ethan R. Peng,[a,b,‡] Anthony M. Burke,[c,‡] David J. Dibble,[c,‡] Chandra B. KC,[c,‡] Reina Kurakake,[a,‡] Panyiming Liu,[a] Robert Lopez,[a] Philip R. Dennison,[b] Alon A. Gorodetsky[a,c,*]

[a]*Department of Materials Science and Engineering, University of California, Irvine, Irvine, CA 92697, USA*
[b]*Department of Chemistry, University of California, Irvine, Irvine, CA 92697, USA*
[c]*Department of Chemical and Biomolecular Engineering, University of California, Irvine, Irvine, CA 92697, USA*
[‡]*These authors contributed equally: Ethan R. Peng, Anthony M. Burke, David J. Dibble, Chandra B. KC, Reina Kurakake.*

[*]*Correspondence to: alon.gorodetsky@uci.edu*

Table of Contents:

I. ^{1}H NMR and ^{13}C NMR Spectroscopy Data S-2

II. UV-Vis Spectroscopy Data S-40

I. ^{1}H NMR and ^{13}C NMR Spectroscopy Data

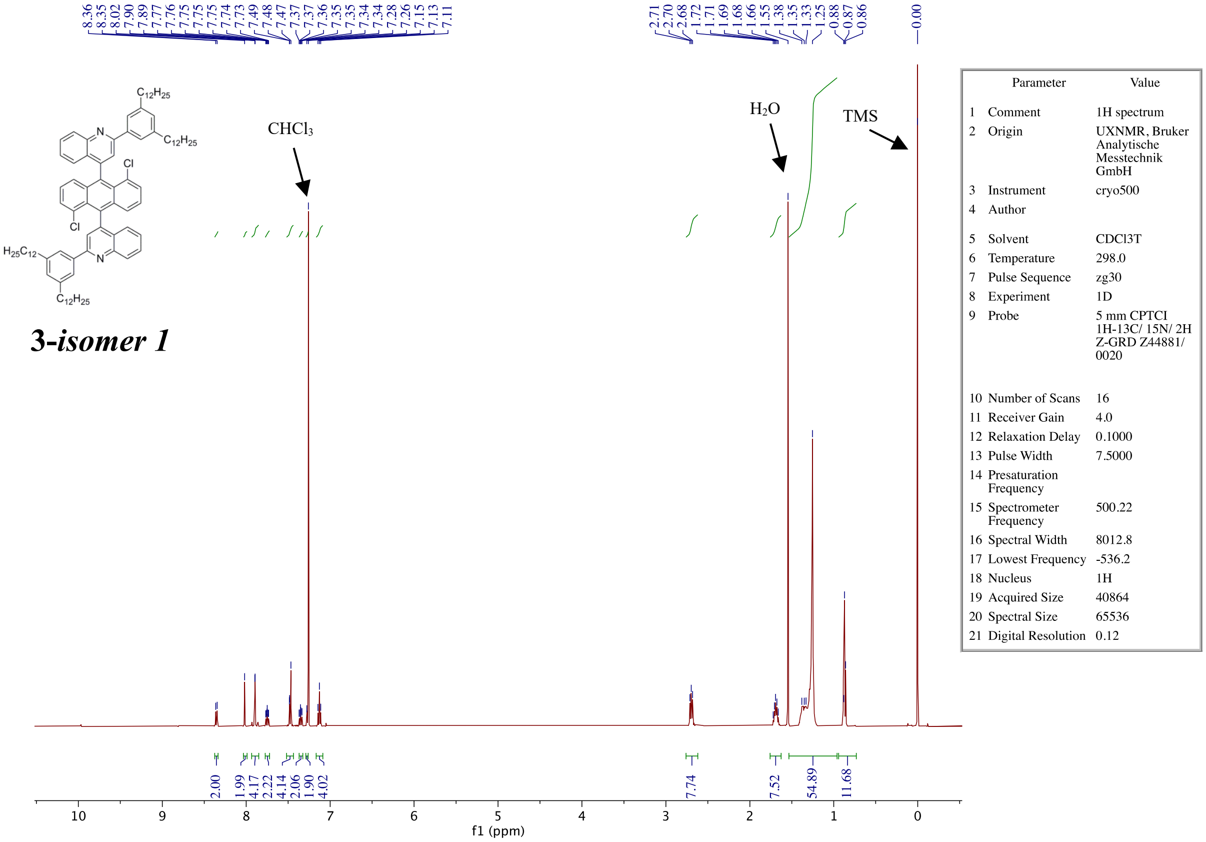

Figure S1. The ^{1}H NMR spectrum obtained for **3-*isomer 1***.

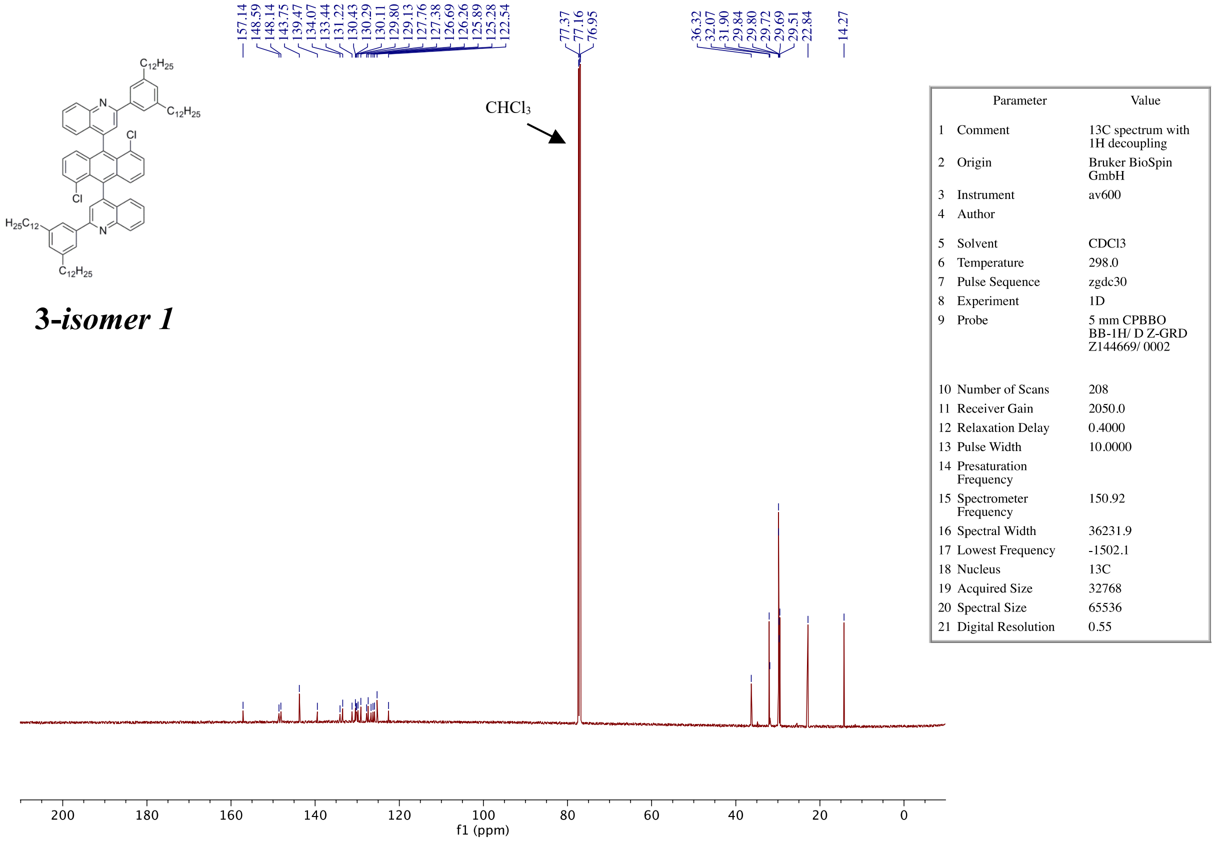

Figure S2. The ^{13}C NMR spectrum obtained for **3-*isomer 1***.

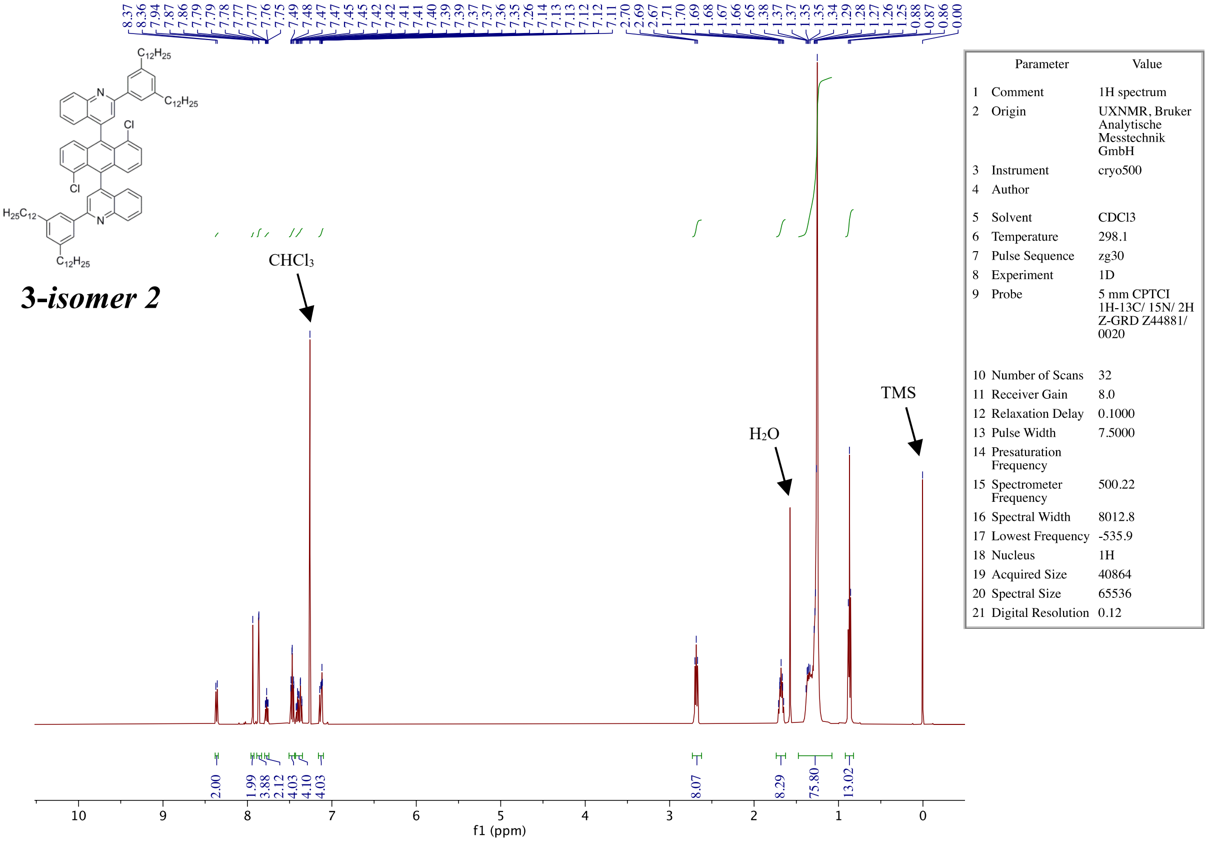

Figure S3. The ^{1}H NMR spectrum obtained for **3-*isomer 2***.

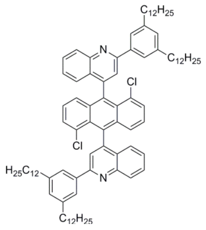

3-isomer 2

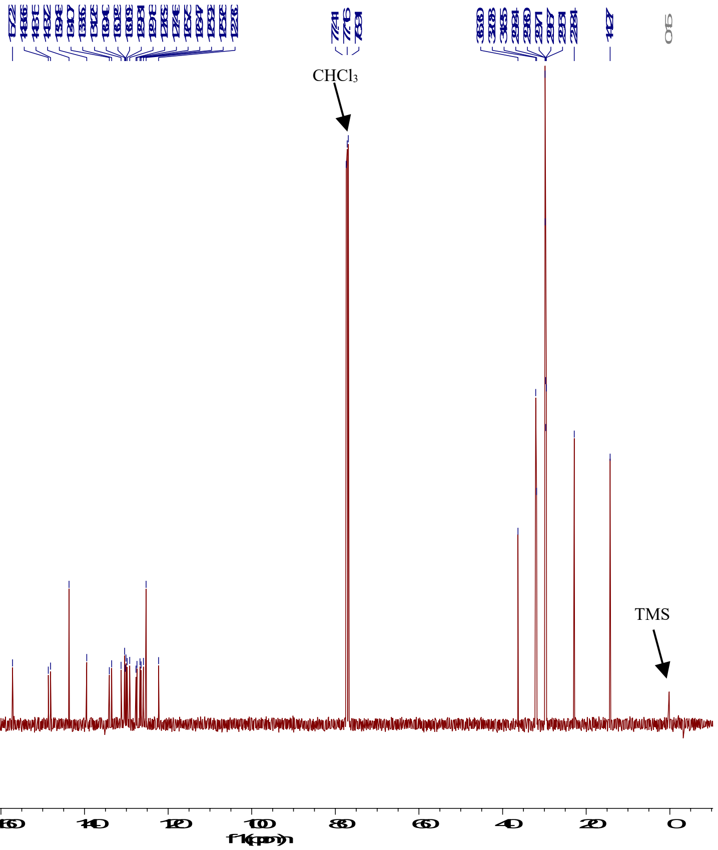

	Parameter	Value
1	Comment	Z-restored spin-echo 13C spectrum with 1H decoupling
2	Origin	UXNMR, Bruker Analytische Messtechnik GmbH
3	Instrument	cryo500
4	Author	
5	Solvent	CDCl3
6	Temperature	298.0
7	Pulse Sequence	SpinEchopg30gp.prd
8	Experiment	1D
9	Probe	5 mm CPTCI 1H-13C/ 15N/ 2H Z-GRD Z44881/ 0020
10	Number of Scans	576
11	Receiver Gain	2896.3
12	Relaxation Delay	0.2500
13	Pulse Width	16.5500
14	Presaturation Frequency	
15	Spectrometer Frequency	125.79
16	Spectral Width	30303.0
17	Lowest Frequency	-1301.8
18	Nucleus	13C
19	Acquired Size	32768
20	Spectral Size	65536
21	Digital Resolution	0.46

Figure S4. The ^{13}C NMR spectrum obtained for ***3-isomer 2***.

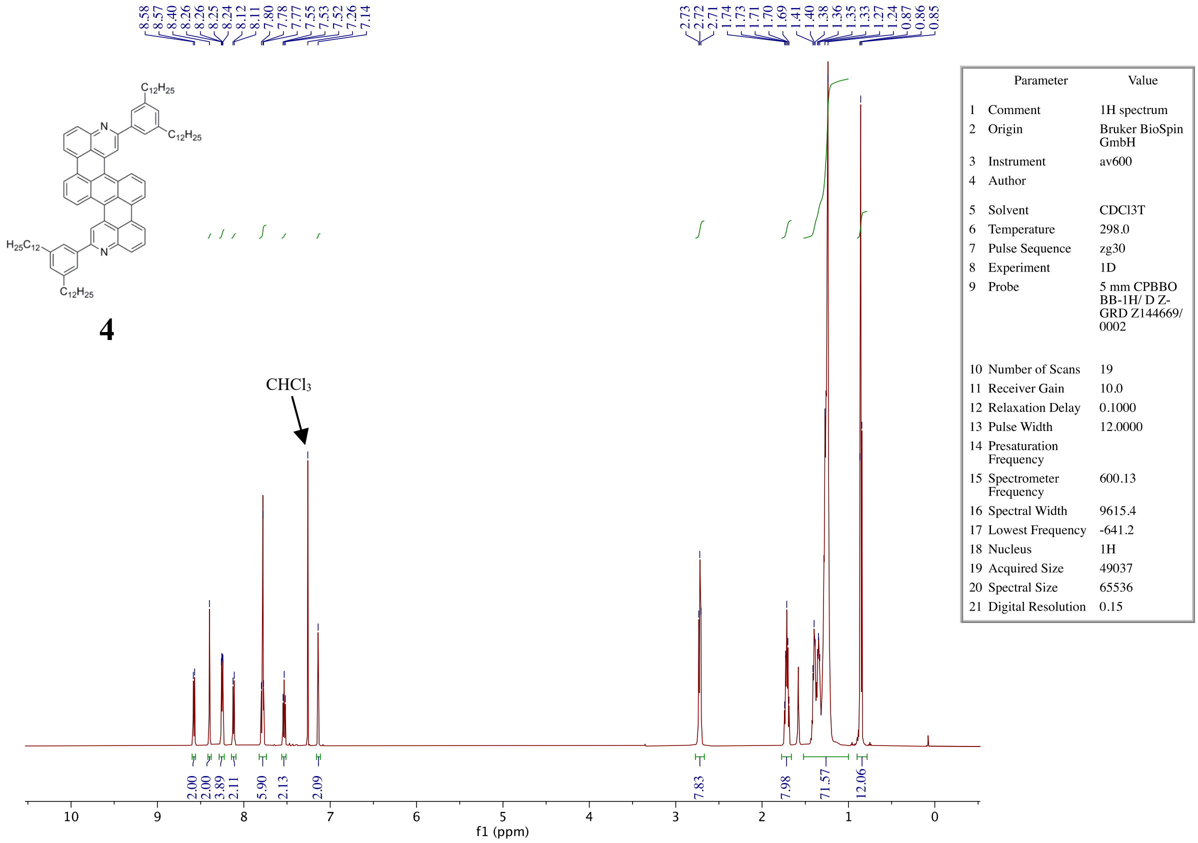

	Parameter	Value
1	Comment	1H spectrum
2	Origin	Bruker BioSpin GmbH
3	Instrument	av600
4	Author	
5	Solvent	CDCl3T
6	Temperature	298.0
7	Pulse Sequence	zg30
8	Experiment	1D
9	Probe	5 mm CPBBO BB-1H/ D Z-GRD Z144669/ 0002
10	Number of Scans	19
11	Receiver Gain	10.0
12	Relaxation Delay	0.1000
13	Pulse Width	12.0000
14	Presaturation Frequency	
15	Spectrometer Frequency	600.13
16	Spectral Width	9615.4
17	Lowest Frequency	-641.2
18	Nucleus	1H
19	Acquired Size	49037
20	Spectral Size	65536
21	Digital Resolution	0.15

Figure S5. The ^{1}H NMR spectrum obtained for **4**.

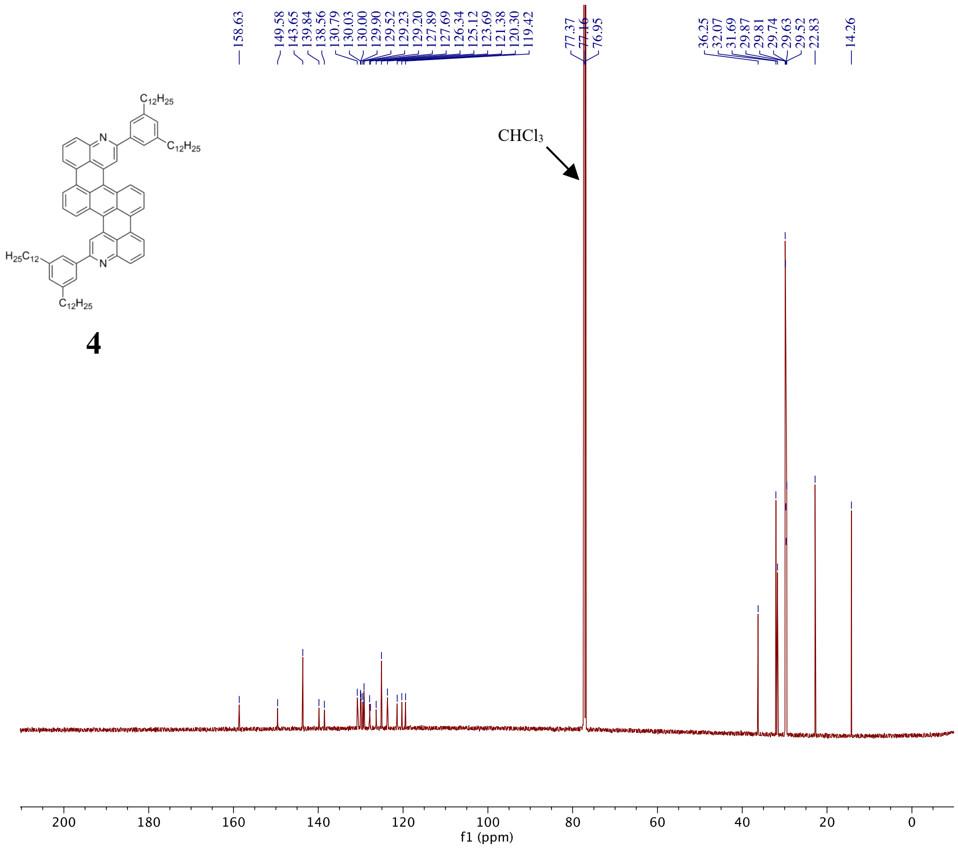

	Parameter	Value
1	Comment	13C spectrum with 1H decoupling
2	Origin	Bruker BioSpin GmbH
3	Instrument	av600
4	Author	
5	Solvent	CDCl3T
6	Temperature	298.0
7	Pulse Sequence	zgdc30
8	Experiment	1D
9	Probe	5 mm CPBBO BB-1H/ D Z-GRD Z144669/ 0002
10	Number of Scans	350
11	Receiver Gain	2050.0
12	Relaxation Delay	0.4000
13	Pulse Width	10.0000
14	Presaturation Frequency	
15	Spectrometer Frequency	150.92
16	Spectral Width	36231.9
17	Lowest Frequency	-1501.6
18	Nucleus	13C
19	Acquired Size	32768
20	Spectral Size	65536
21	Digital Resolution	0.55

Figure S6. The ^{13}C NMR spectrum obtained for **4**.

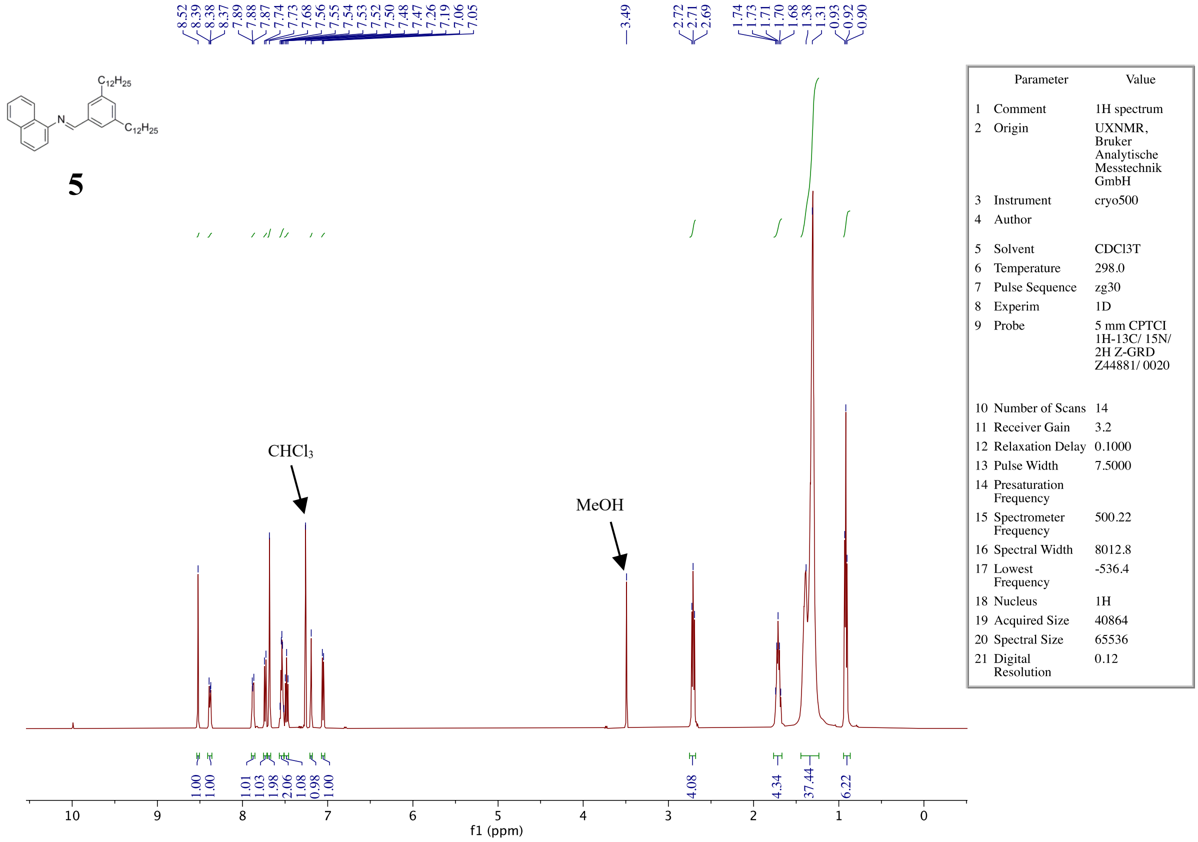

Parameter		Value
1	Comment	1H spectrum
2	Origin	UXNMR, Bruker Analytische Messtechnik GmbH
3	Instrument	cryo500
4	Author	
5	Solvent	CDCl3T
6	Temperature	298.0
7	Pulse Sequence	zg30
8	Experim	1D
9	Probe	5 mm CPTCI 1H-13C/ 15N/ 2H Z-GRD Z44881/ 0020
10	Number of Scans	14
11	Receiver Gain	3.2
12	Relaxation Delay	0.1000
13	Pulse Width	7.5000
14	Presaturation Frequency	
15	Spectrometer Frequency	500.22
16	Spectral Width	8012.8
17	Lowest Frequency	-536.4
18	Nucleus	1H
19	Acquired Size	40864
20	Spectral Size	65536
21	Digital Resolution	0.12

Figure S7. The ^{1}H NMR spectrum obtained for **5**.

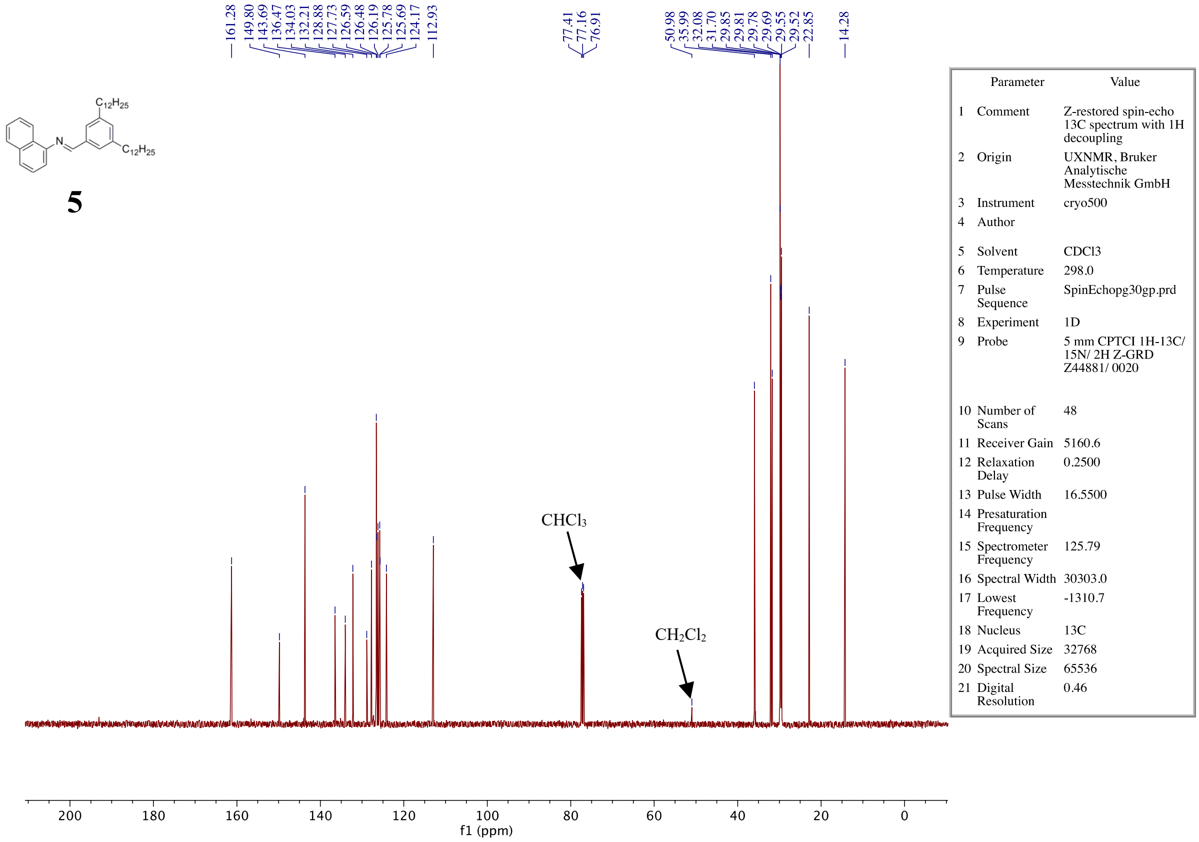

	Parameter	Value
1	Comment	Z-restored spin-echo 13C spectrum with 1H decoupling
2	Origin	UXNMR, Bruker Analytische Messtechnik GmbH
3	Instrument	cryo500
4	Author	
5	Solvent	CDCl3
6	Temperature	298.0
7	Pulse Sequence	SpinEchopg30gp.prd
8	Experiment	1D
9	Probe	5 mm CPTCI 1H-13C/ 15N/ 2H Z-GRD Z44881/ 0020
10	Number of Scans	48
11	Receiver Gain	5160.6
12	Relaxation Delay	0.2500
13	Pulse Width	16.5500
14	Presaturation Frequency	
15	Spectrometer Frequency	125.79
16	Spectral Width	30303.0
17	Lowest Frequency	-1310.7
18	Nucleus	13C
19	Acquired Size	32768
20	Spectral Size	65536
21	Digital Resolution	0.46

Figure S8. The ^{13}C NMR spectrum obtained for **5**.

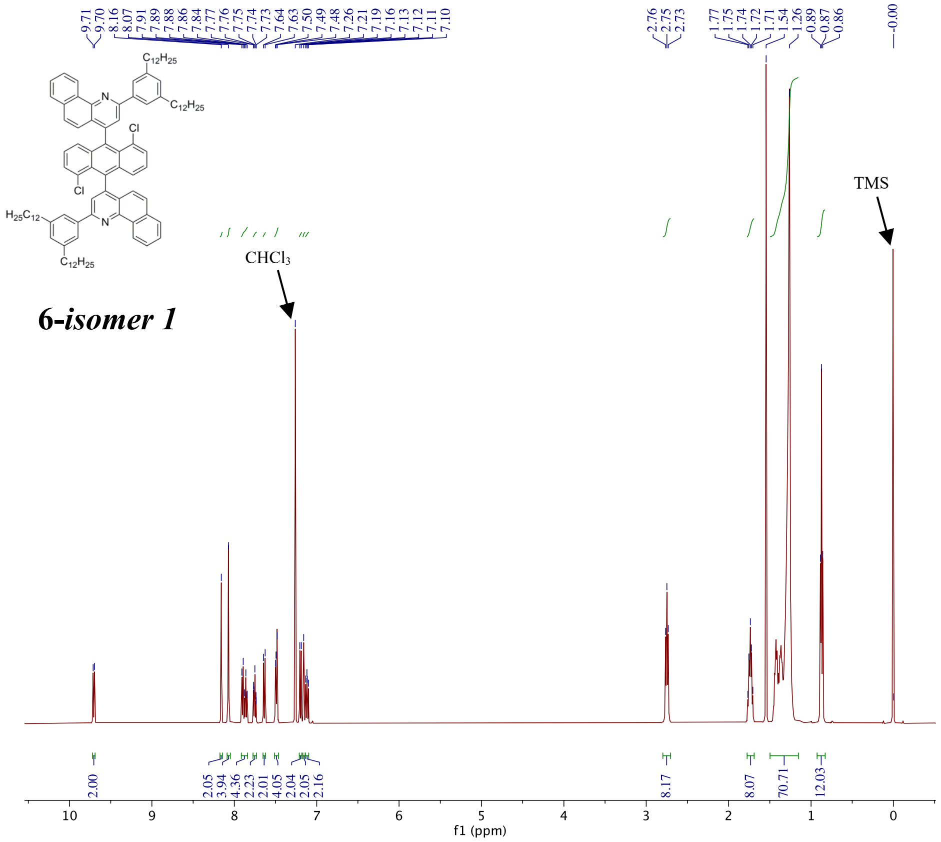

Parameter		Value
1	Comment	1H spectrum
2	Origin	Bruker Analytik GmbH
3	Instrument	cryo500
4	Author	
5	Solvent	CDCl3T
6	Temperature	298.0
7	Pulse Sequence	zg30
8	Experiment	1D
9	Probe	5 mm CPTCI 1H-13C/ 15N/ 2H Z-GRD Z44881/ 0020
10	Number of Scans	32
11	Receiver Gain	7.1
12	Relaxation Delay	0.1000
13	Pulse Width	7.5000
14	Presaturation Frequency	
15	Spectrometer Frequency	500.22
16	Spectral Width	8012.8
17	Lowest Frequency	-535.8
18	Nucleus	1H
19	Acquired Size	40864
20	Spectral Size	65536
21	Digital Resolution	0.12

Figure S9. The ^{1}H NMR spectrum obtained for **6-*isomer 1***.

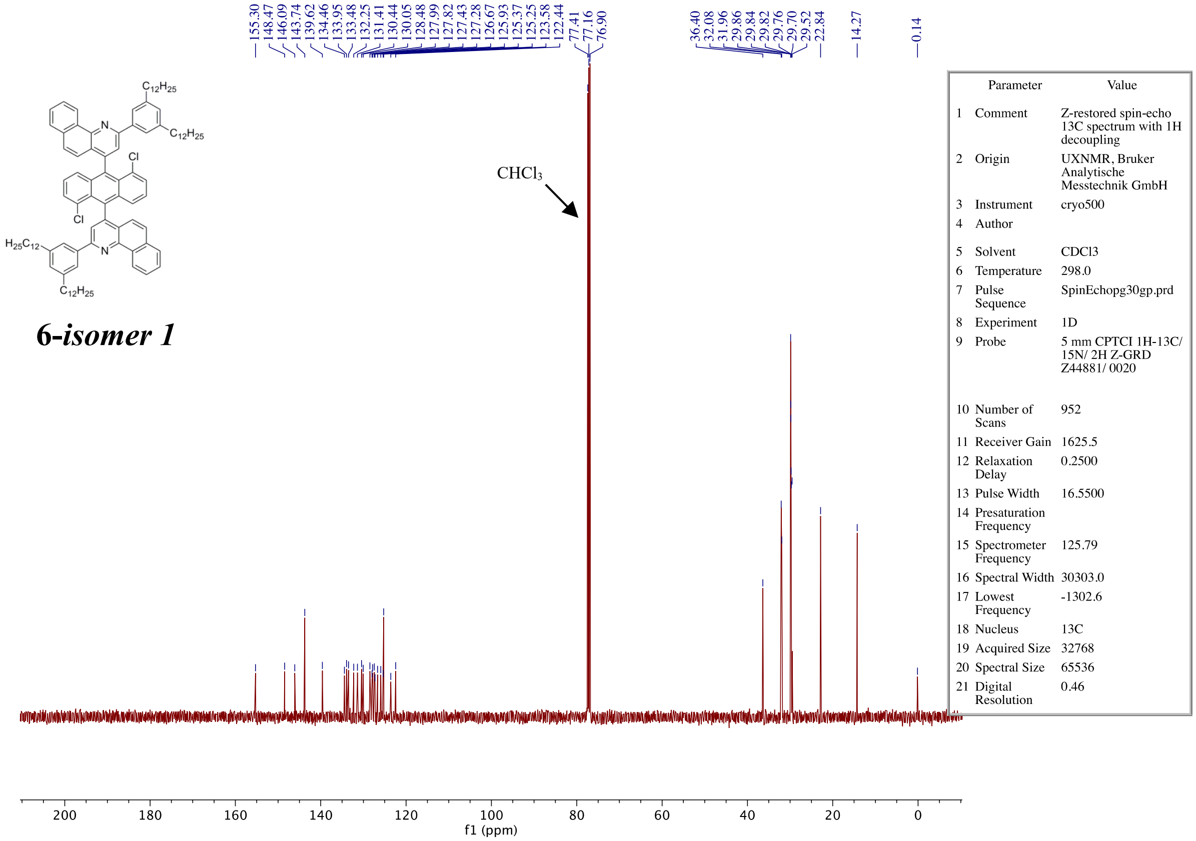

Figure S10. The ^{13}C NMR spectrum obtained for ***6-isomer 1***.

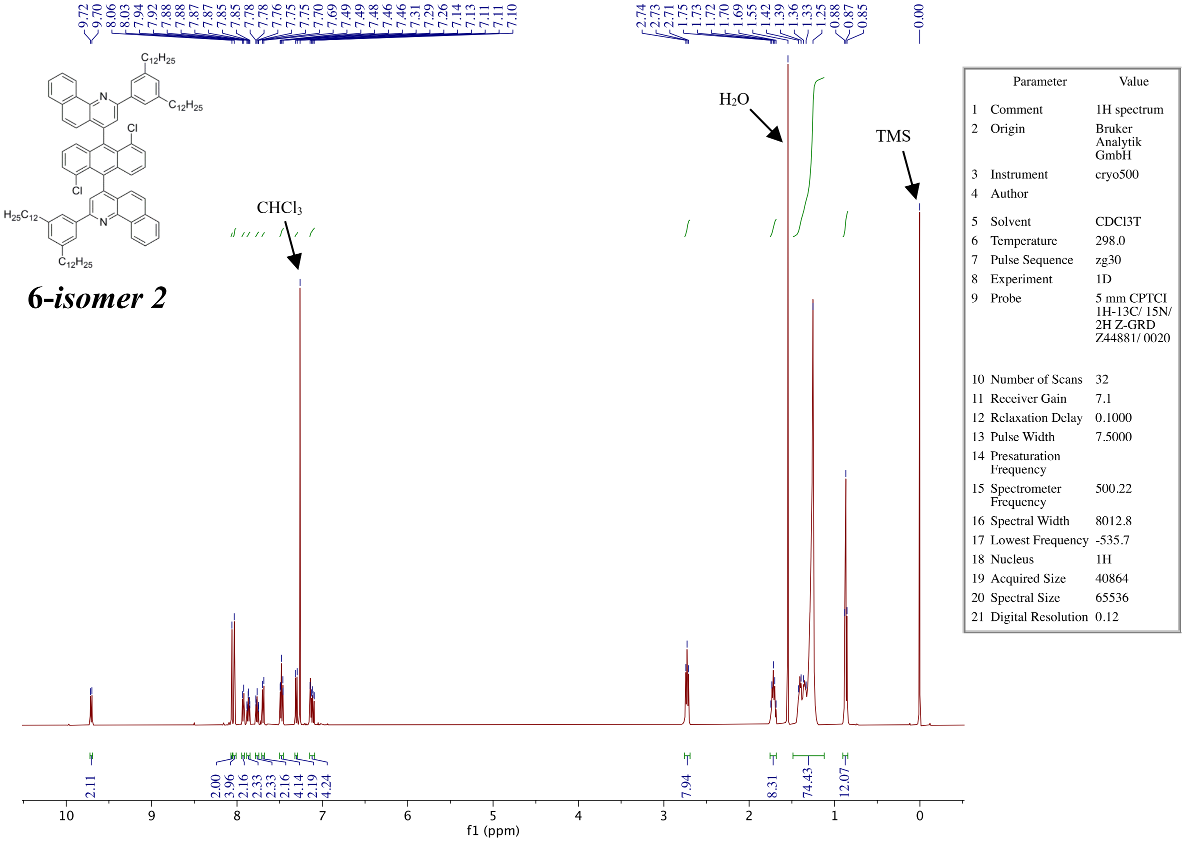

Figure S11. The ^{1}H NMR spectrum obtained for ***6-isomer 2***.

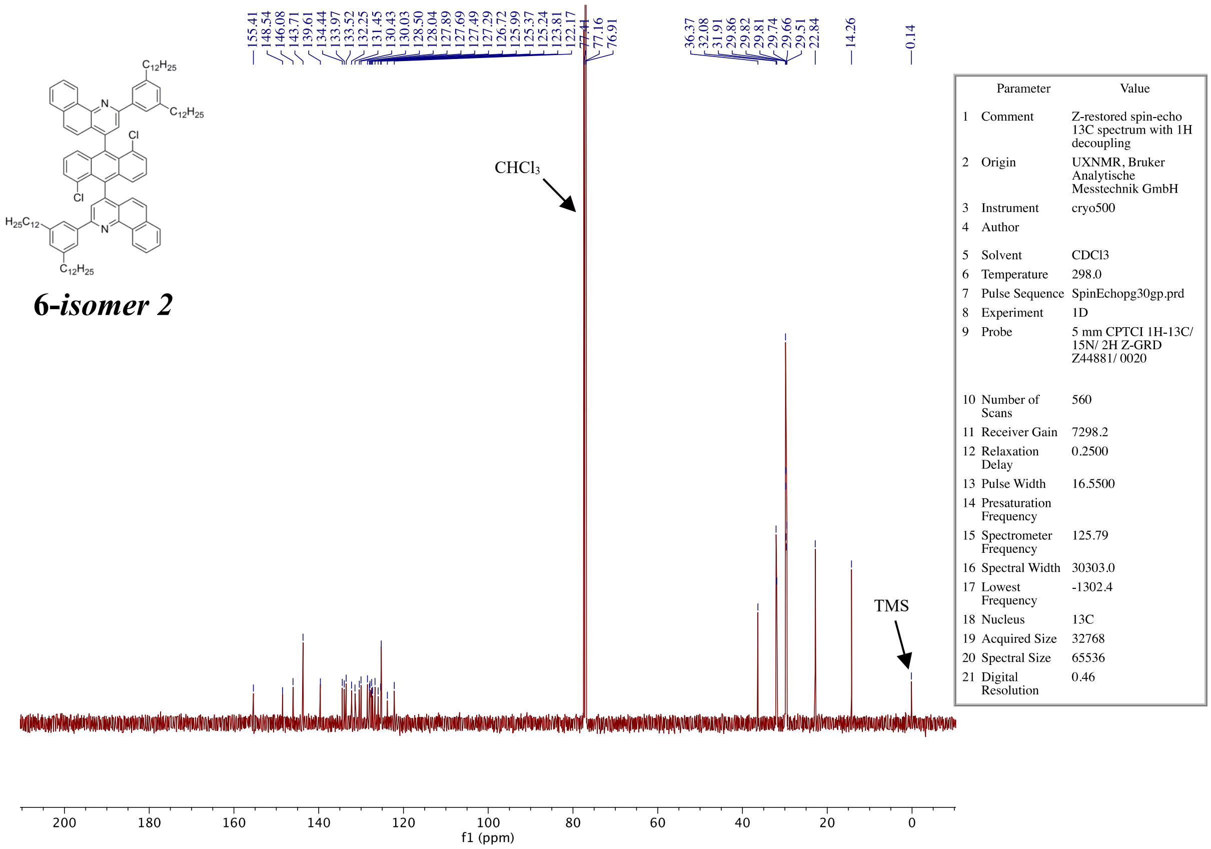

Figure S12. The ^{13}C NMR spectrum obtained for ***6-isomer 2***.

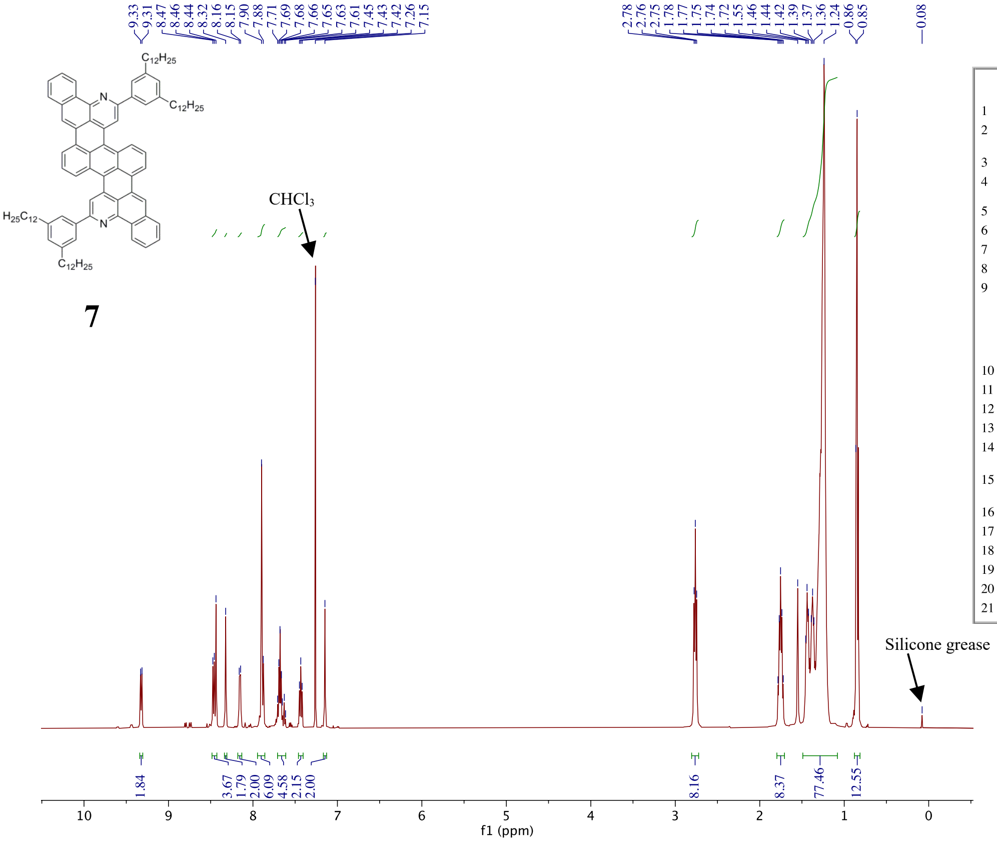

	Parameter	Value
1	Comment	1H spectrum
2	Origin	Bruker Analytik GmbH
3	Instrument	cryo500
4	Author	
5	Solvent	CDCl3T
6	Temperature	298.0
7	Pulse Sequence	zg30
8	Experiment	1D
9	Probe	5 mm CPTCI 1H-13C/ 15N/ 2H Z-GRD Z44881/ 0020
10	Number of Scans	12
11	Receiver Gain	5.7
12	Relaxation Delay	0.1000
13	Pulse Width	7.5000
14	Presaturation Frequency	
15	Spectrometer Frequency	500.22
16	Spectral Width	8012.8
17	Lowest Frequency	-536.4
18	Nucleus	1H
19	Acquired Size	40864
20	Spectral Size	65536
21	Digital Resolution	0.12

Figure S13. The ^{1}H NMR spectrum obtained for **7**.

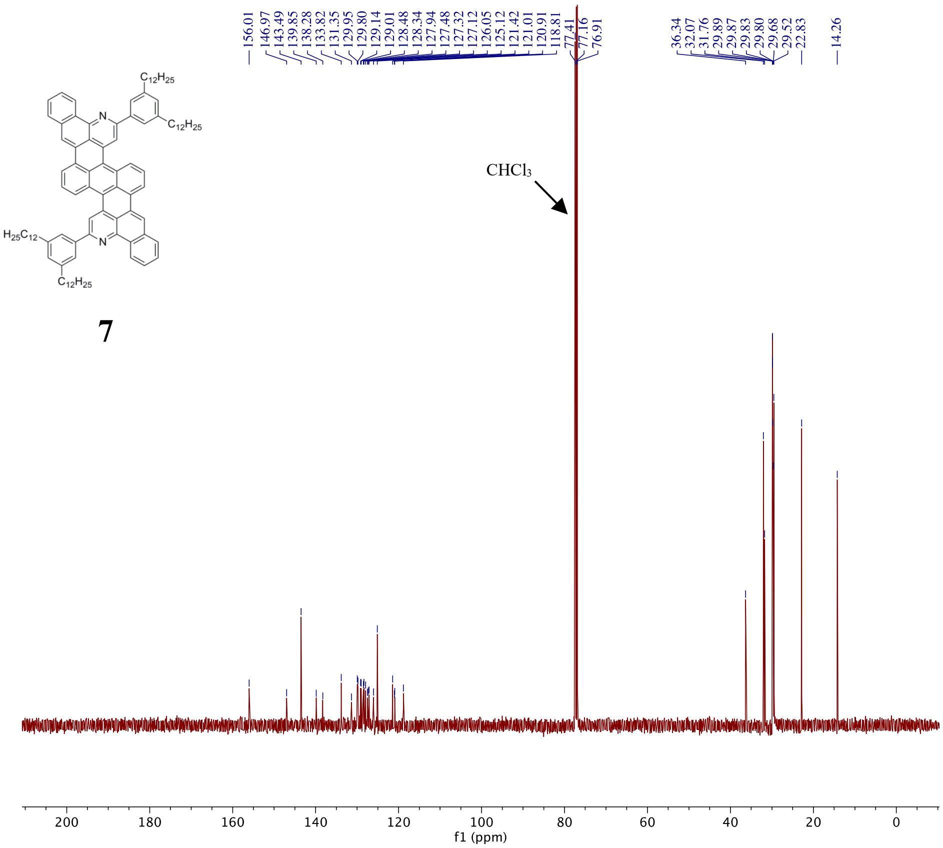

	Parameter	Value
1	Comment	Z-restored spin-echo 13C spectrum with 1H decoupling
2	Origin	UXNMR, Bruker Analytische Messtechnik GmbH
3	Instrument	cryo500
4	Author	
5	Solvent	CDCl3
6	Temperature	298.0
7	Pulse Sequence	SpinEchopg30gp.prd
8	Experiment	1D
9	Probe	5 mm CPTCI 1H-13C/ 15N/ 2H Z-GRD Z44881/ 0020
10	Number of Scans	516
11	Receiver Gain	6502.0
12	Relaxation Delay	0.2500
13	Pulse Width	16.5500
14	Presaturation Frequency	
15	Spectrometer Frequency	125.79
16	Spectral Width	30303.0
17	Lowest Frequency	-1304.7
18	Nucleus	13C
19	Acquired Size	32768
20	Spectral Size	65536
21	Digital Resolution	0.46

Figure S14. The ^{13}C NMR spectrum obtained for **7**.

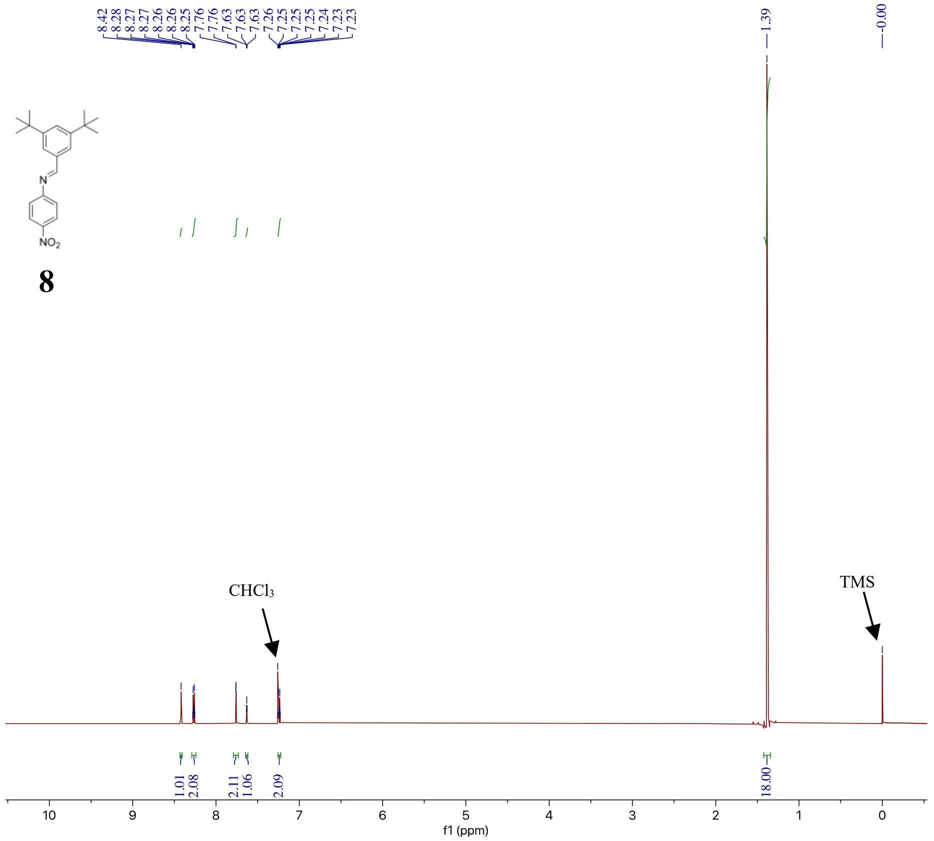

	Parameter	Value
1	Comment	1H Spectrum
2	Origin	Bruker BioSpin GmbH
3	Instrument	av600
4	Author	
5	Solvent	CDCl3
6	Temperature	298.0
7	Pulse Sequence	zg30
8	Experiment	1D
9	Probe	5 mm CPBBO BB-1H/ D Z-GRD Z144669/ 0002
10	Number of Scans	8
11	Receiver Gain	45.2
12	Relaxation Delay	0.1000
13	Pulse Width	12.0000
14	Presaturation Frequency	
15	Spectrometer Frequency	600.13
16	Spectral Width	9615.4
17	Lowest Frequency	-643.5
18	Nucleus	1H
19	Acquired Size	49037
20	Spectral Size	65536
21	Digital Resolution	0.15

Figure S15. The ^{1}H NMR spectrum obtained for **8.**

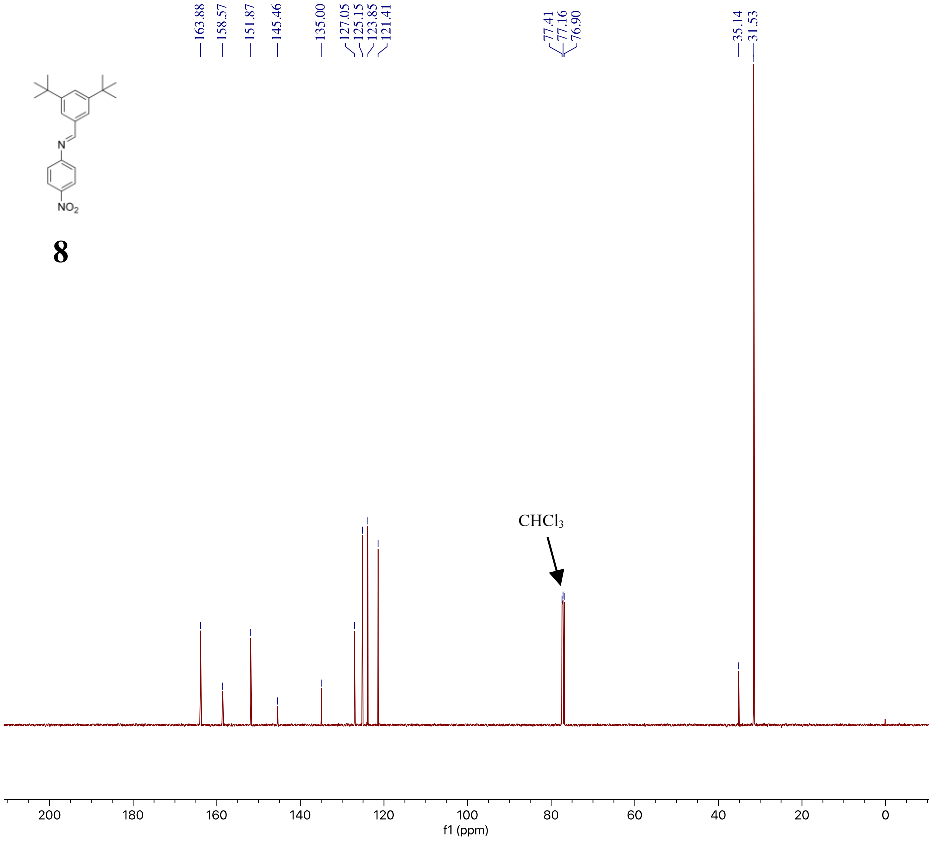

	Parameter	Value
1	Comment	Z-restored spin-echo 13C spectrum with 1H decoupling
2	Origin	Bruker Analytik GmbH
3	Instrument	cryo500
4	Author	
5	Solvent	CDCl3
6	Temperature	298.2
7	Pulse Sequence	SpinEchopg30gp2.prd
8	Experiment	1D
9	Probe	5 mm CPTCI 1H-13C/ 15N/ 2H Z-GRD Z44881/ 0020
10	Number of Scans	1024
11	Receiver Gain	8192.0
12	Relaxation Delay	0.2500
13	Pulse Width	16.5500
14	Presaturation Frequency	
15	Spectrometer Frequency	125.79
16	Spectral Width	30303.0
17	Lowest Frequency	-1303.8
18	Nucleus	13C
19	Acquired Size	32768
20	Spectral Size	65536
21	Digital Resolution	0.46

Figure S16. The ^{13}C NMR spectrum obtained for **8.**

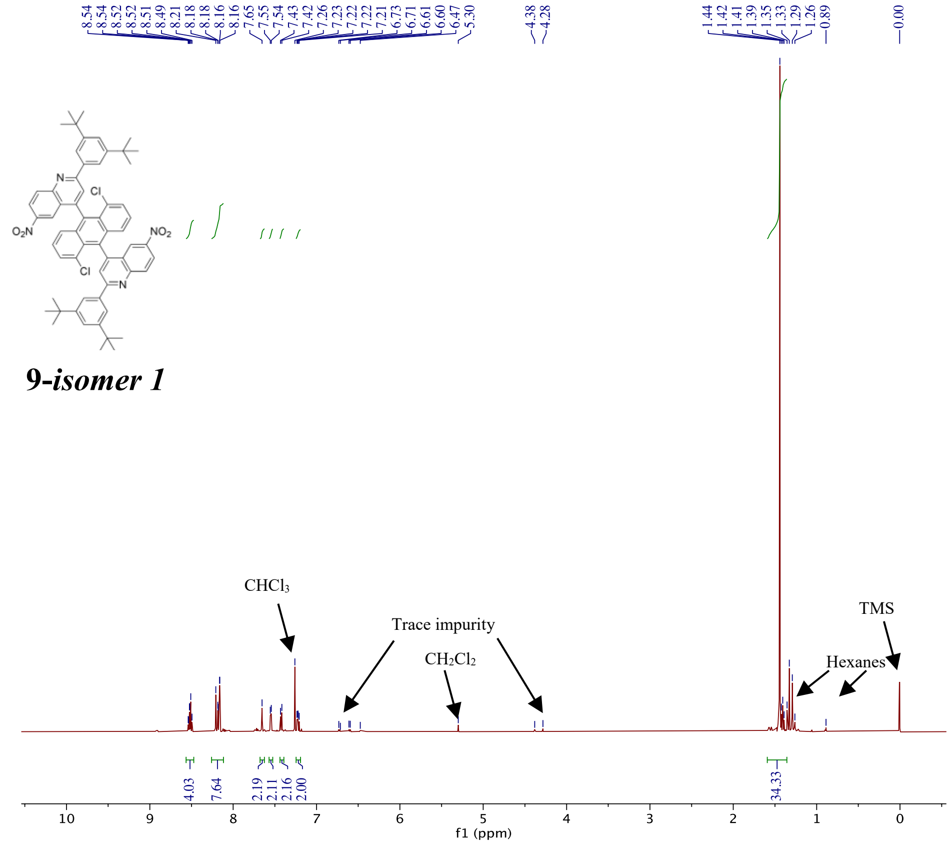

	Parameter	Value
1	Comment	1H spectrum
2	Origin	Bruker BioSpin GmbH
3	Instrument	av600
4	Author	
5	Solvent	CDCl3
6	Temperature	298.0
7	Pulse Sequence	zg30
8	Experiment	1D
9	Probe	5 mm CPBBO BB-1H/ D Z-GRD Z144669/ 0002
10	Number of Scans	8
11	Receiver Gain	10.0
12	Relaxation Delay	0.1000
13	Pulse Width	9.5000
14	Presaturation Frequency	
15	Spectrometer Frequency	600.13
16	Spectral Width	9615.4
17	Lowest Frequency	-644.6
18	Nucleus	1H
19	Acquired Size	49037
20	Spectral Size	65536
21	Digital Resolution	0.15

Figure S17. The ^{1}H NMR spectrum obtained for ***9-isomer 1***.

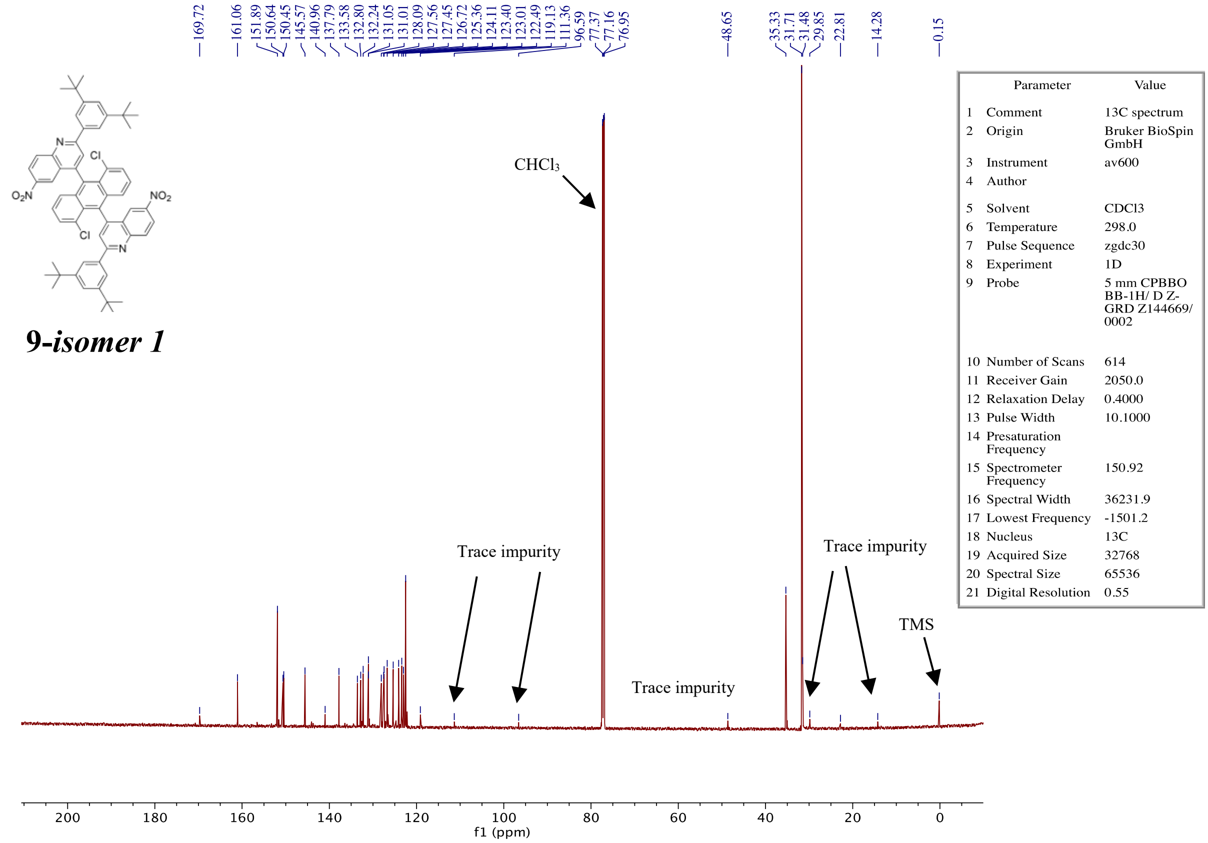

	Parameter	Value
1	Comment	13C spectrum
2	Origin	Bruker BioSpin GmbH
3	Instrument	av600
4	Author	
5	Solvent	CDCl3
6	Temperature	298.0
7	Pulse Sequence	zgdc30
8	Experiment	1D
9	Probe	5 mm CPBBO BB-1H/ D Z-GRD Z144669/ 0002
10	Number of Scans	614
11	Receiver Gain	2050.0
12	Relaxation Delay	0.4000
13	Pulse Width	10.1000
14	Presaturation Frequency	
15	Spectrometer Frequency	150.92
16	Spectral Width	36231.9
17	Lowest Frequency	-1501.2
18	Nucleus	13C
19	Acquired Size	32768
20	Spectral Size	65536
21	Digital Resolution	0.55

Figure S18. The ^{13}C NMR spectrum obtained for ***9-isomer 1***.

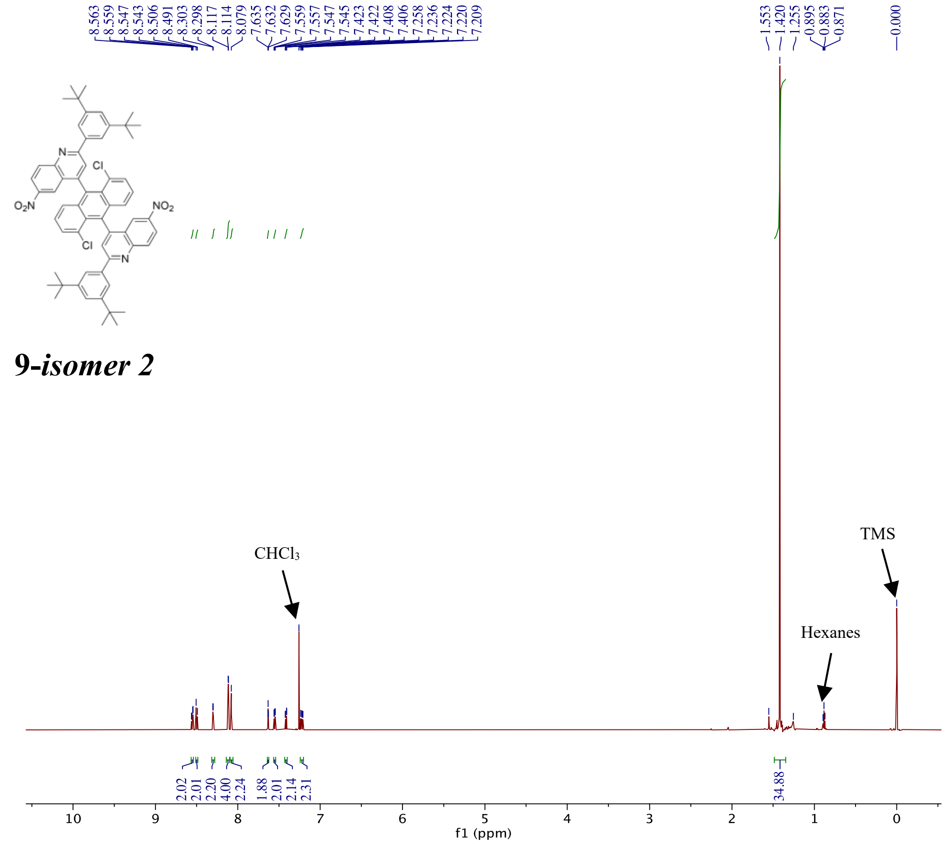

Parameter		Value
1	Comment	1H spectrum
2	Origin	Bruker BioSpin GmbH
3	Instrument	av600
4	Author	
5	Solvent	CDCl3
6	Temperature	298.0
7	Pulse Sequence	zg30
8	Experiment	1D
9	Probe	5 mm CPBBO BB-1H/ D Z-GRD Z144669/ 0002
10	Number of Scans	8
11	Receiver Gain	10.0
12	Relaxation Delay	0.1000
13	Pulse Width	9.5000
14	Presaturation Frequency	
15	Spectrometer Frequency	600.13
16	Spectral Width	9615.4
17	Lowest Frequency	-644.0
18	Nucleus	1H
19	Acquired Size	49037
20	Spectral Size	65536
21	Digital Resolution	0.15

Figure S19. The ^{1}H NMR spectrum obtained for ***9-isomer 2***.

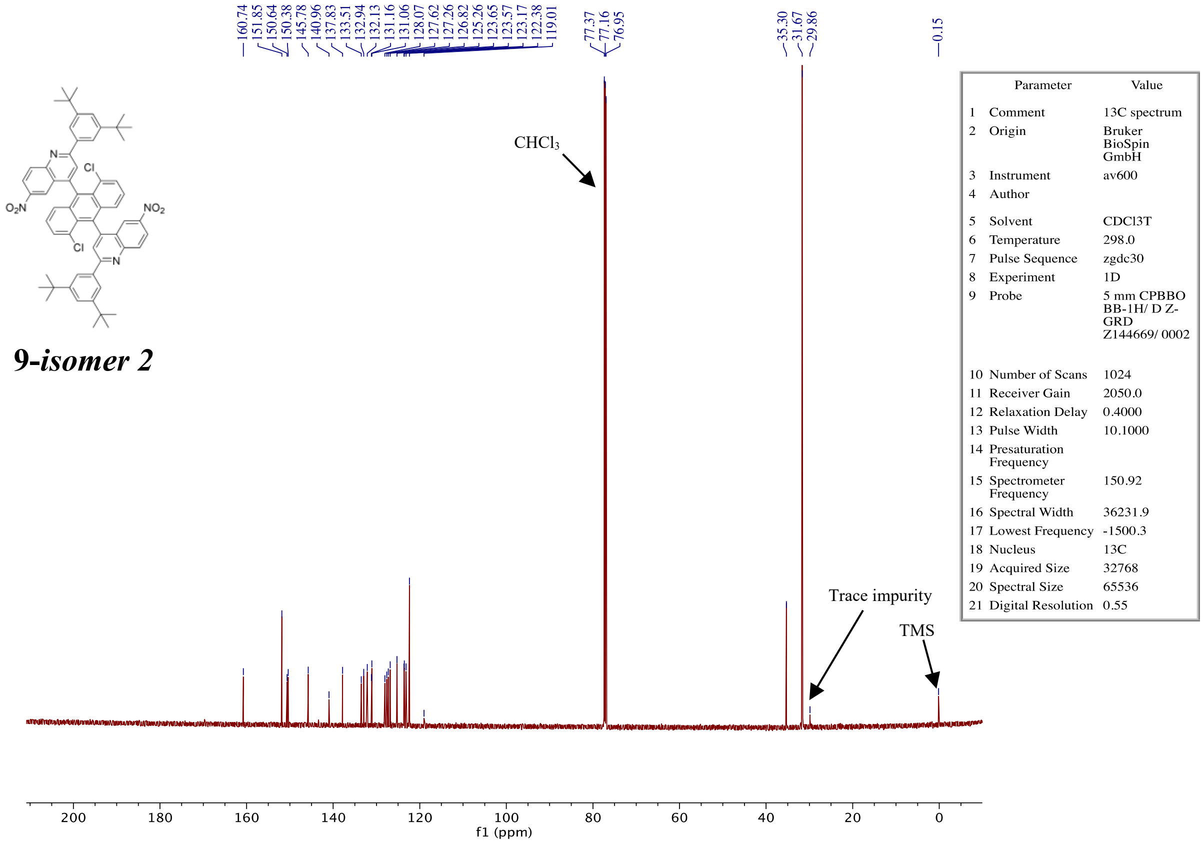

	Parameter	Value
1	Comment	13C spectrum
2	Origin	Bruker BioSpin GmbH
3	Instrument	av600
4	Author	
5	Solvent	CDCl3T
6	Temperature	298.0
7	Pulse Sequence	zgdc30
8	Experiment	1D
9	Probe	5 mm CPBBO BB-1H/ D Z-GRD Z144669/ 0002
10	Number of Scans	1024
11	Receiver Gain	2050.0
12	Relaxation Delay	0.4000
13	Pulse Width	10.1000
14	Presaturation Frequency	
15	Spectrometer Frequency	150.92
16	Spectral Width	36231.9
17	Lowest Frequency	-1500.3
18	Nucleus	13C
19	Acquired Size	32768
20	Spectral Size	65536
21	Digital Resolution	0.55

Figure S20. The ^{13}C NMR spectrum obtained for **9-*isomer 2***.

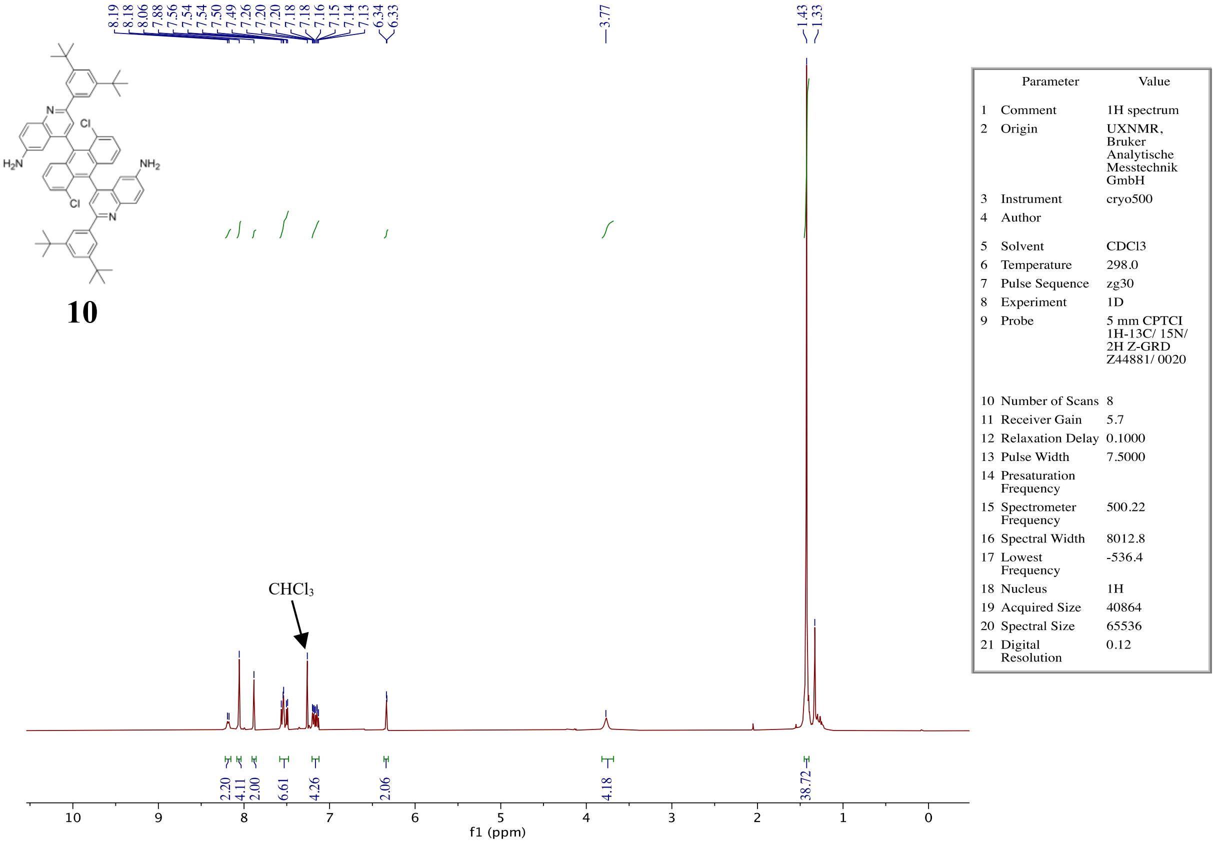

	Parameter	Value
1	Comment	1H spectrum
2	Origin	UXNMR, Bruker Analytische Messtechnik GmbH
3	Instrument	cryo500
4	Author	
5	Solvent	CDCl3
6	Temperature	298.0
7	Pulse Sequence	zg30
8	Experiment	1D
9	Probe	5 mm CPTCI 1H-13C/ 15N/ 2H Z-GRD Z44881/ 0020
10	Number of Scans	8
11	Receiver Gain	5.7
12	Relaxation Delay	0.1000
13	Pulse Width	7.5000
14	Presaturation Frequency	
15	Spectrometer Frequency	500.22
16	Spectral Width	8012.8
17	Lowest Frequency	-536.4
18	Nucleus	1H
19	Acquired Size	40864
20	Spectral Size	65536
21	Digital Resolution	0.12

Figure S21. The ^{1}H NMR spectrum obtained for **10**.

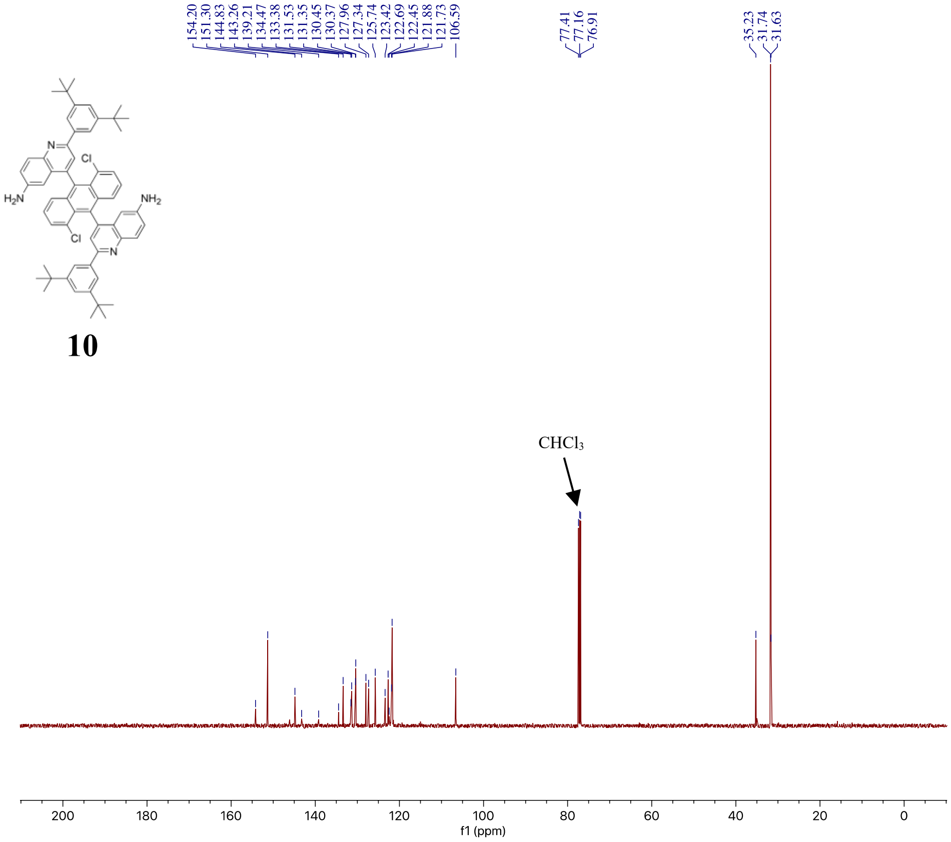

	Parameter	Value
1	Comment	Z-restored spin-echo 13C spectrum with 1H decoupling
2	Origin	UXNMR, Bruker Analytische Messtechnik GmbH
3	Instrument	cryo500
4	Author	
5	Solvent	CDCl3
6	Temperature	298.0
7	Pulse Sequence	SpinEchopg30gp.prd
8	Experiment	1D
9	Probe	5 mm CPTCI 1H-13C/ 15N/ 2H Z-GRD Z44881/ 0020
10	Number of Scans	792
11	Receiver Gain	10321.3
12	Relaxation Delay	0.2500
13	Pulse Width	16.5500
14	Presaturation Frequency	
15	Spectrometer Frequency	125.79
16	Spectral Width	30303.0
17	Lowest Frequency	-1307.0
18	Nucleus	13C
19	Acquired Size	32768
20	Spectral Size	65536
21	Digital Resolution	0.46

Figure S22. The ^{13}C NMR spectrum obtained for **10**.

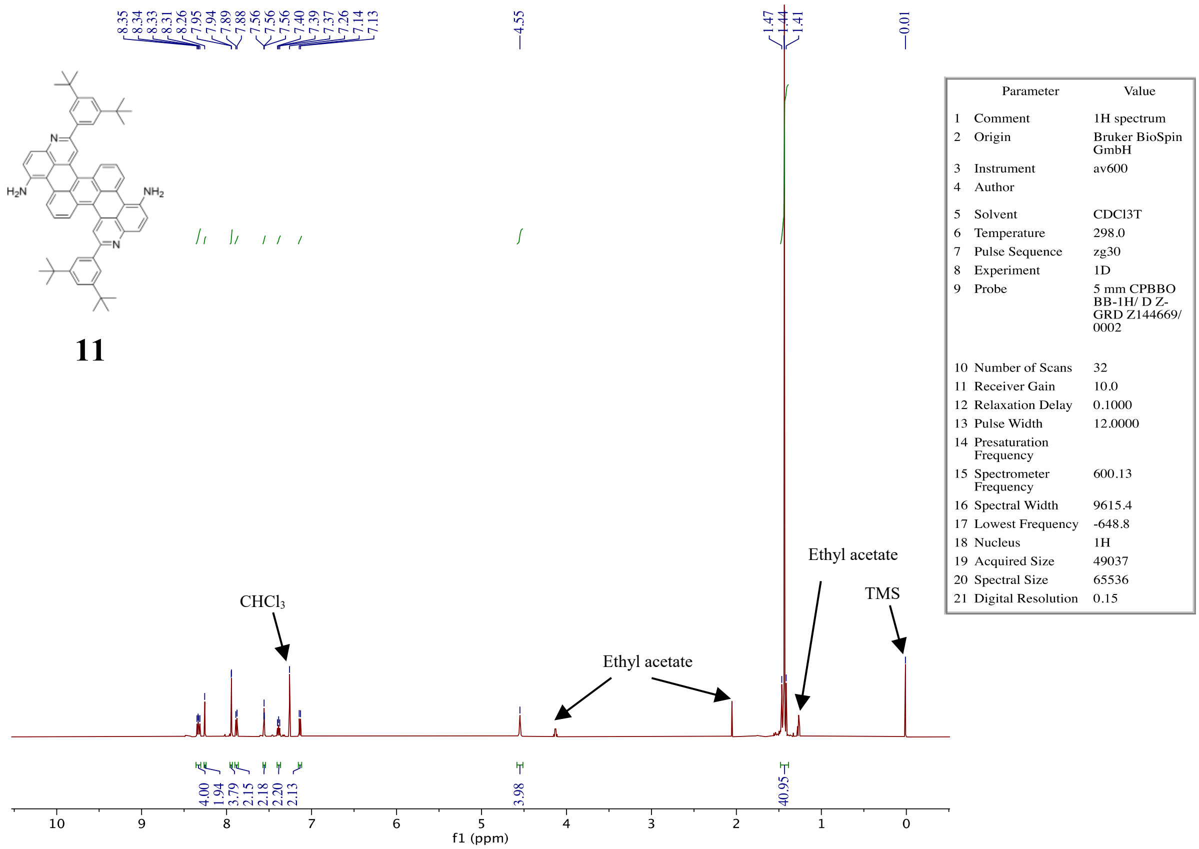

Parameter		Value
1	Comment	1H spectrum
2	Origin	Bruker BioSpin GmbH
3	Instrument	av600
4	Author	
5	Solvent	CDCl3T
6	Temperature	298.0
7	Pulse Sequence	zg30
8	Experiment	1D
9	Probe	5 mm CPBBO BB-1H/ D Z-GRD Z144669/ 0002
10	Number of Scans	32
11	Receiver Gain	10.0
12	Relaxation Delay	0.1000
13	Pulse Width	12.0000
14	Presaturation Frequency	
15	Spectrometer Frequency	600.13
16	Spectral Width	9615.4
17	Lowest Frequency	-648.8
18	Nucleus	1H
19	Acquired Size	49037
20	Spectral Size	65536
21	Digital Resolution	0.15

Figure S23. The ^{1}H NMR spectrum obtained for **11**.

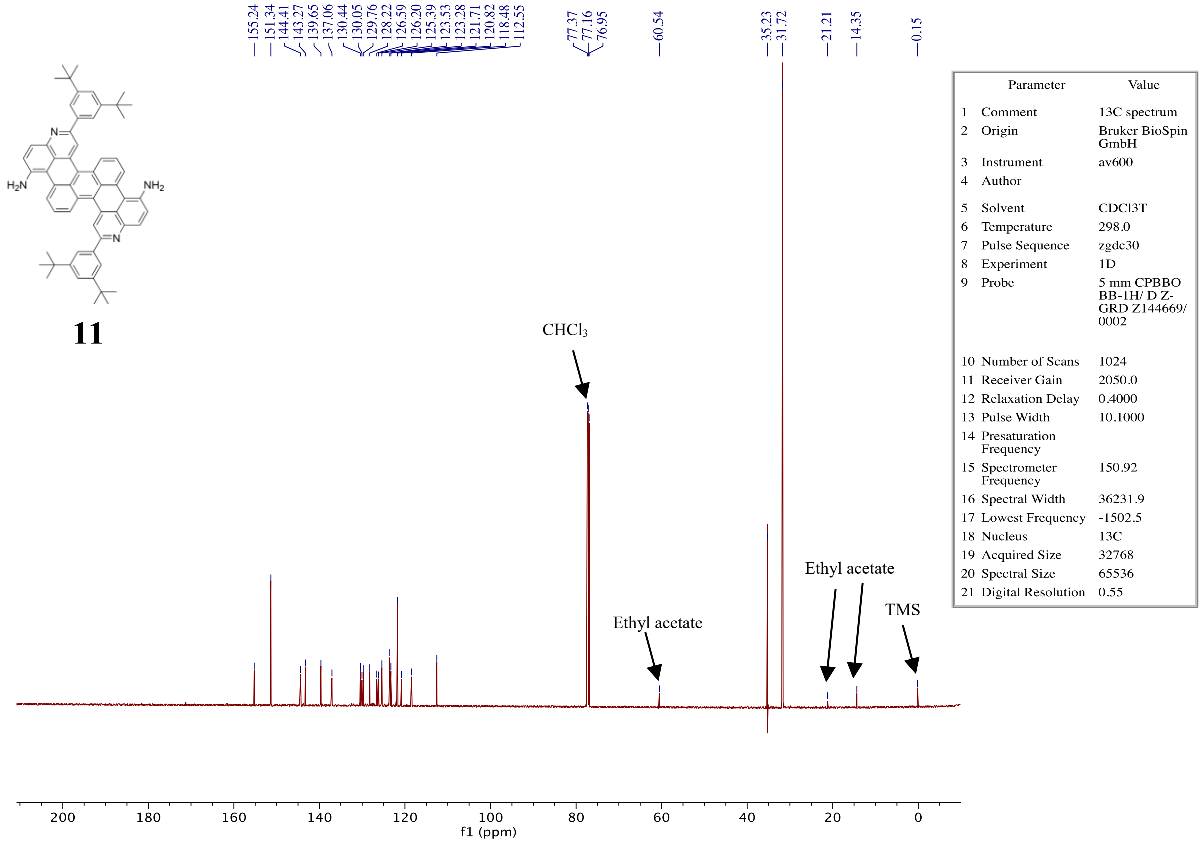

Figure S24. The ^{13}C NMR spectrum obtained for **11.**

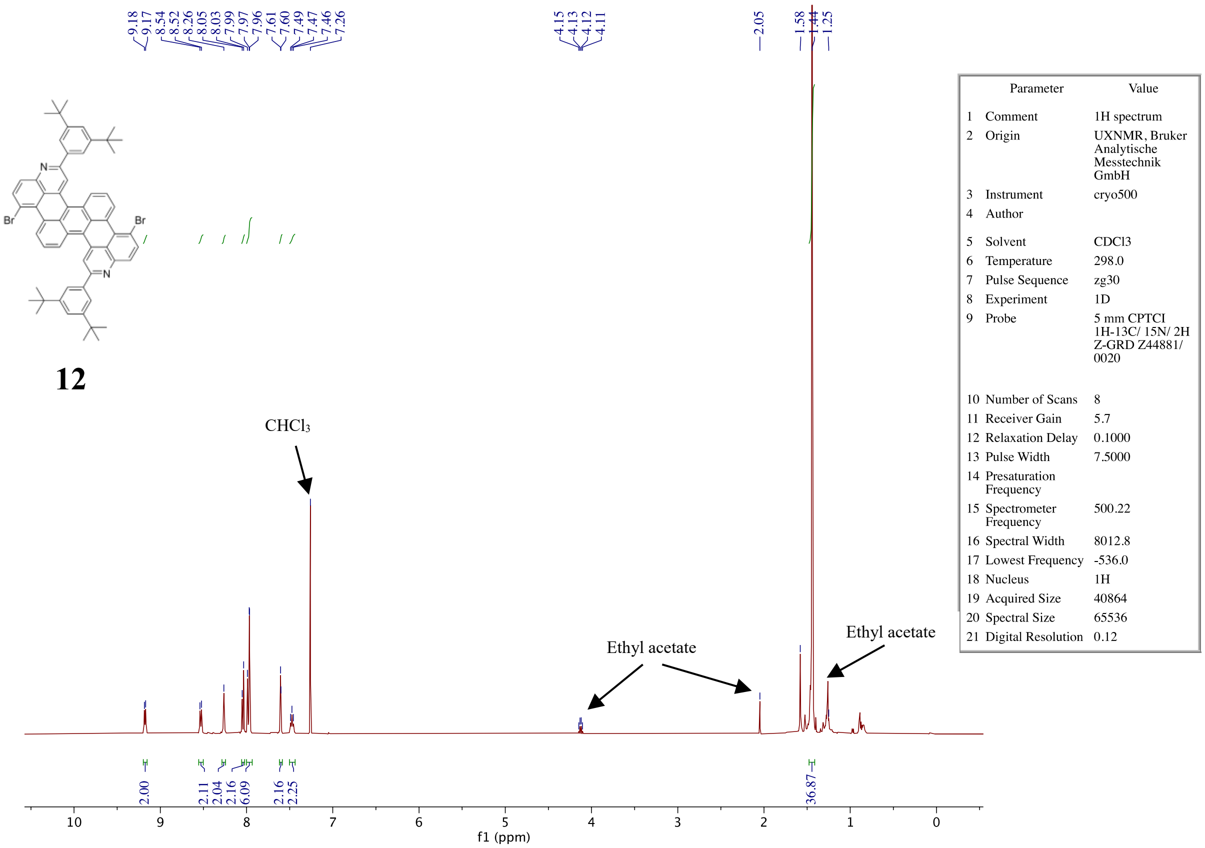

Figure S25. The ^{1}H NMR spectrum obtained for **12**.

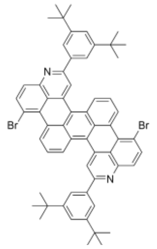

12

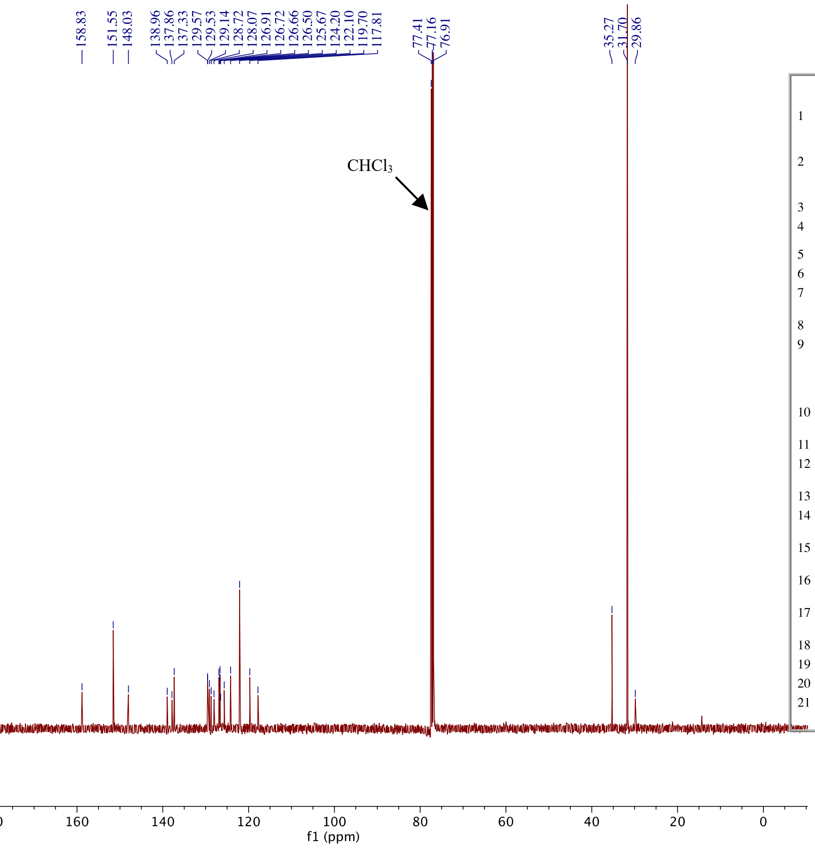

	Parameter	Value
1	Comment	Z-restored spin-echo 13C spectrum with 1H decoupling
2	Origin	UXNMR, Bruker Analytische Messtechnik GmbH
3	Instrument	cryo500
4	Author	
5	Solvent	CDCl3
6	Temperature	298.0
7	Pulse Sequence	SpinEchopg30gp.prd
8	Experiment	1D
9	Probe	5 mm CPTCI 1H-13C/ 15N/ 2H Z-GRD Z44881/ 0020
10	Number of Scans	1488
11	Receiver Gain	3649.1
12	Relaxation Delay	0.2500
13	Pulse Width	16.5500
14	Presaturation Frequency	
15	Spectrometer Frequency	125.79
16	Spectral Width	30303.0
17	Lowest Frequency	-1302.4
18	Nucleus	13C
19	Acquired Size	32768
20	Spectral Size	65536
21	Digital Resolution	0.46

Figure S26. The ^{13}C NMR spectrum obtained for **12**.

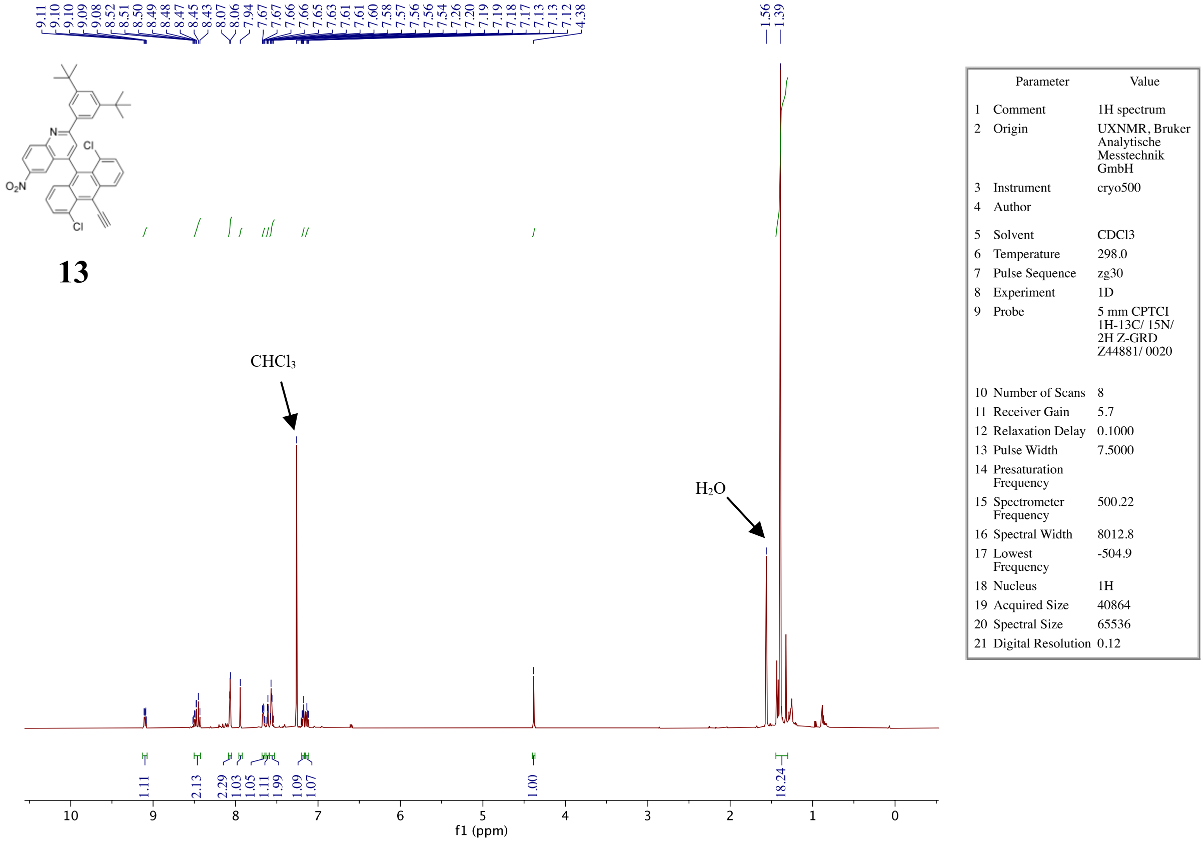

Figure S27. The ^{1}H NMR spectrum obtained for **13**.

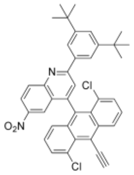

13

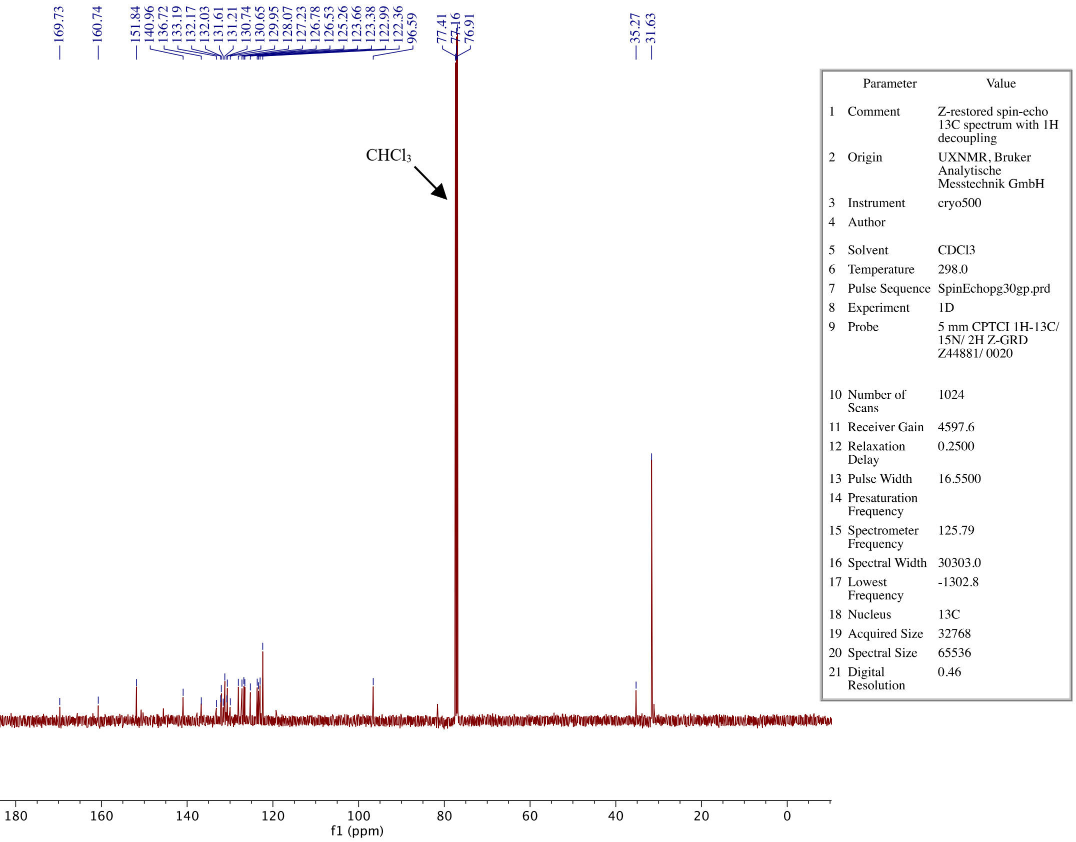

Figure S28. The ^{13}C NMR spectrum obtained for **13**.

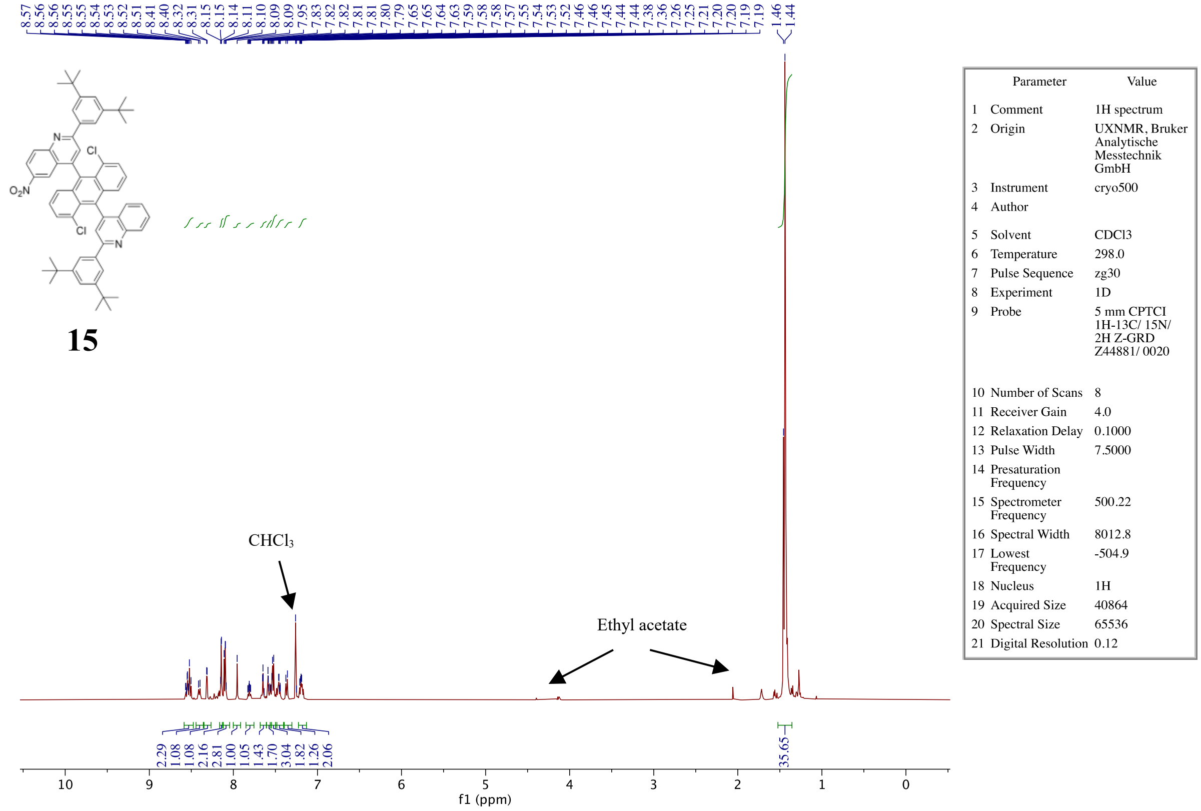

Figure S29. The ^{1}H NMR spectrum obtained for **15**.

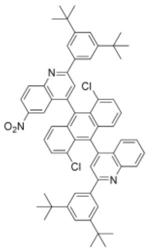

15

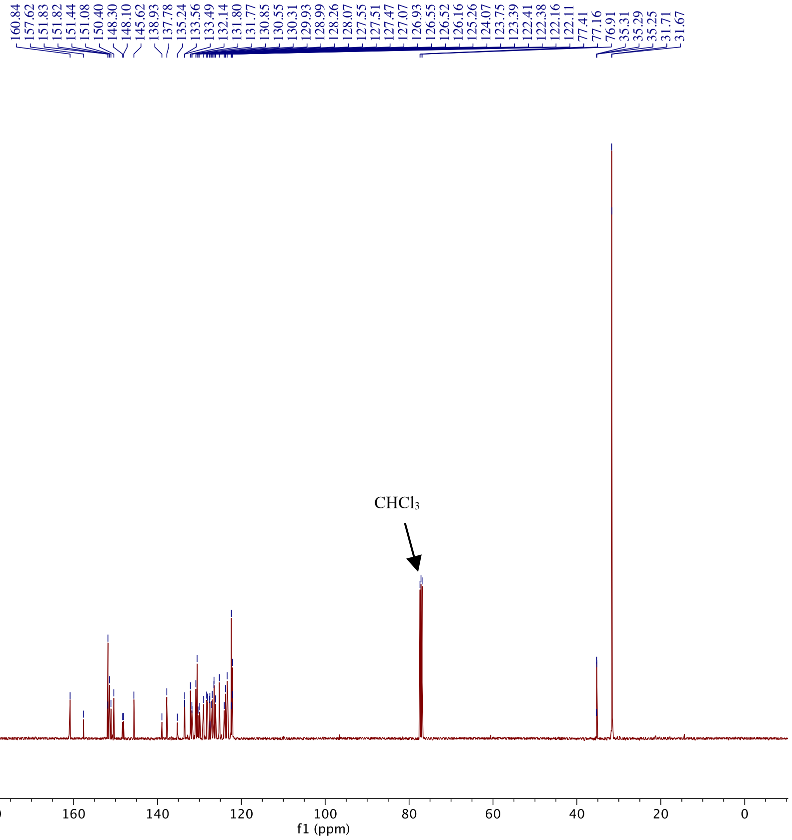

	Parameter	Value
1	Comment	Z-restored spin-echo 13C spectrum with 1H decoupling
2	Origin	UXNMR, Bruker Analytische Messtechnik GmbH
3	Instrument	cryo500
4	Author	
5	Solvent	CDCl3
6	Temperature	298.0
7	Pulse Sequence	SpinEchopg30gp.prd
8	Experiment	1D
9	Probe	5 mm CPTCI 1H-13C/ 15N/ 2H Z-GRD Z44881/ 0020
10	Number of Scans	976
11	Receiver Gain	2580.3
12	Relaxation Delay	0.2500
13	Pulse Width	16.5500
14	Presaturation Frequency	
15	Spectrometer Frequency	125.79
16	Spectral Width	30303.0
17	Lowest Frequency	-1306.9
18	Nucleus	13C
19	Acquired Size	32768
20	Spectral Size	65536
21	Digital Resolution	0.46

Figure S30. The ^{13}C NMR spectrum obtained for **15**.

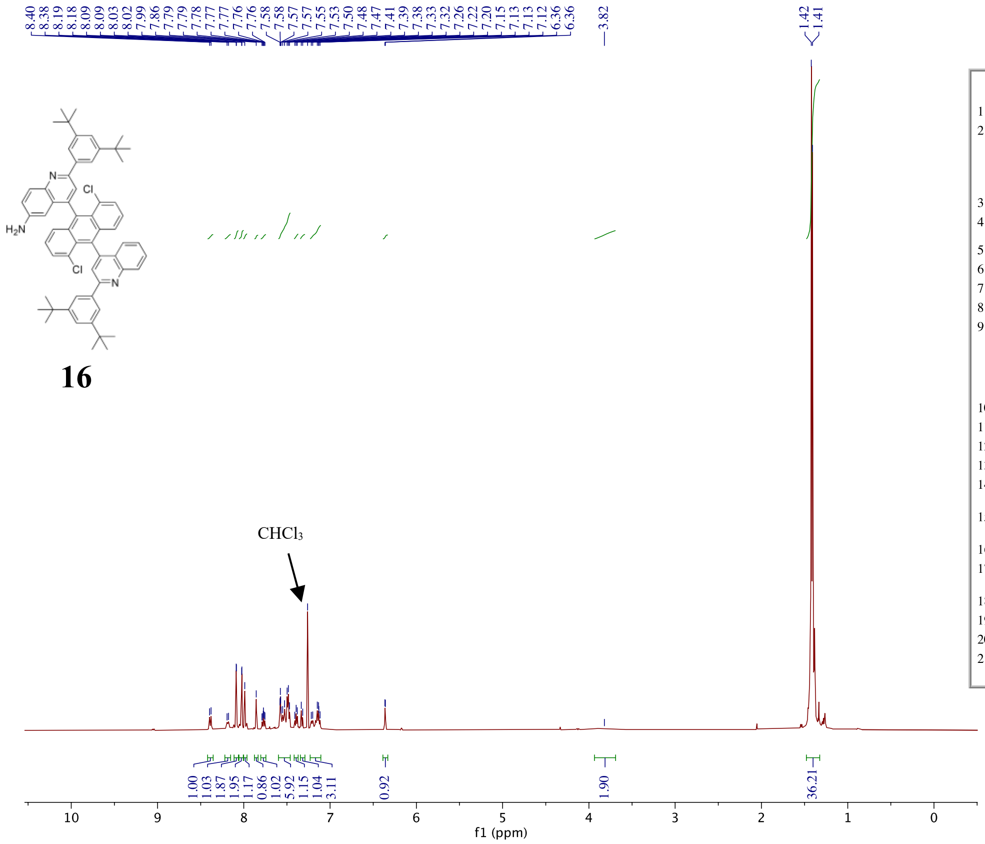

	Parameter	Value
1	Comment	1H spectrum
2	Origin	UXNMR, Bruker Analytische Messtechnik GmbH
3	Instrument	cryo500
4	Author	
5	Solvent	CDCl3
6	Temperature	298.0
7	Pulse Sequence	zg30
8	Experiment	1D
9	Probe	5 mm CPTCI 1H-13C/ 15N/ 2H Z-GRD Z44881/ 0020
10	Number of Scans	8
11	Receiver Gain	3.2
12	Relaxation Delay	0.1000
13	Pulse Width	7.5000
14	Presaturation Frequency	
15	Spectrometer Frequency	500.22
16	Spectral Width	8012.8
17	Lowest Frequency	-504.9
18	Nucleus	1H
19	Acquired Size	40864
20	Spectral Size	65536
21	Digital Resolution	0.12

Figure S31. The ^{1}H NMR spectrum obtained for **16**.

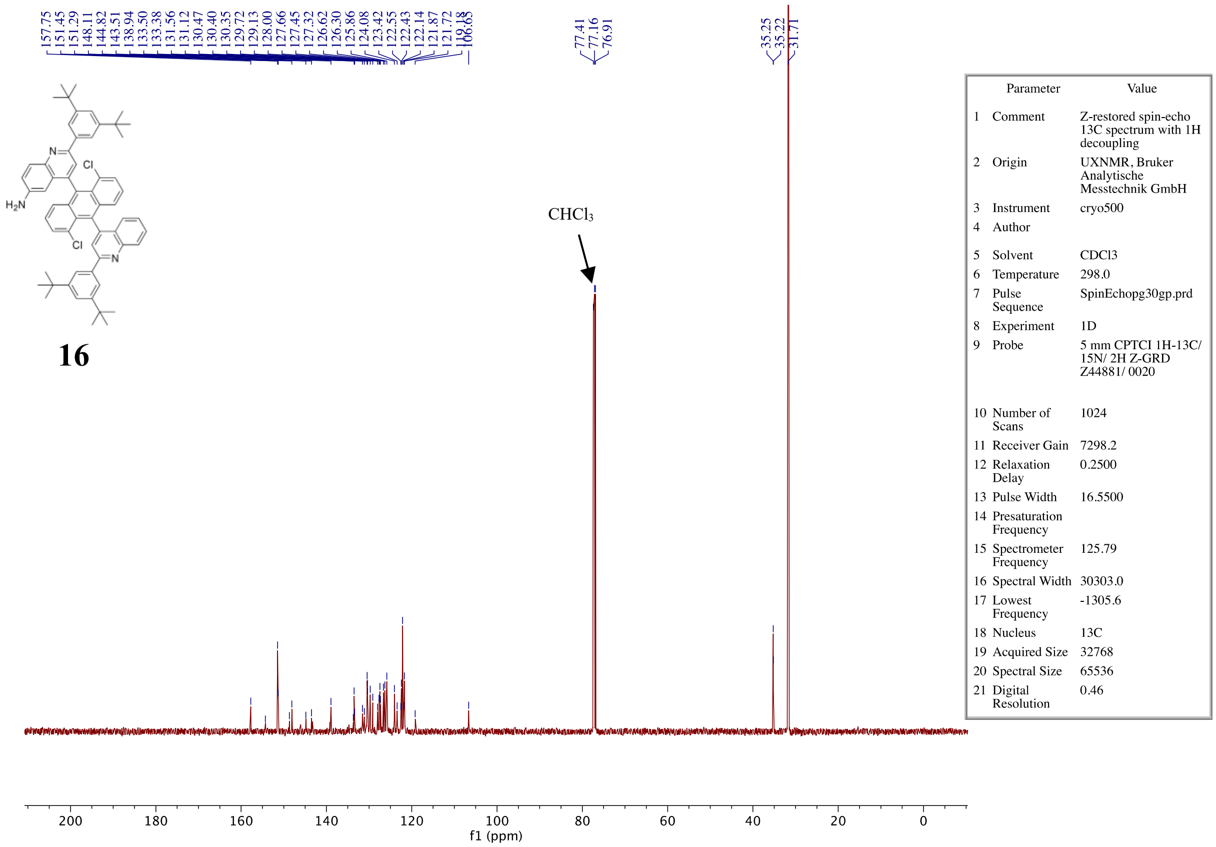

Figure S32. The ^{13}C NMR spectrum obtained for **16**.

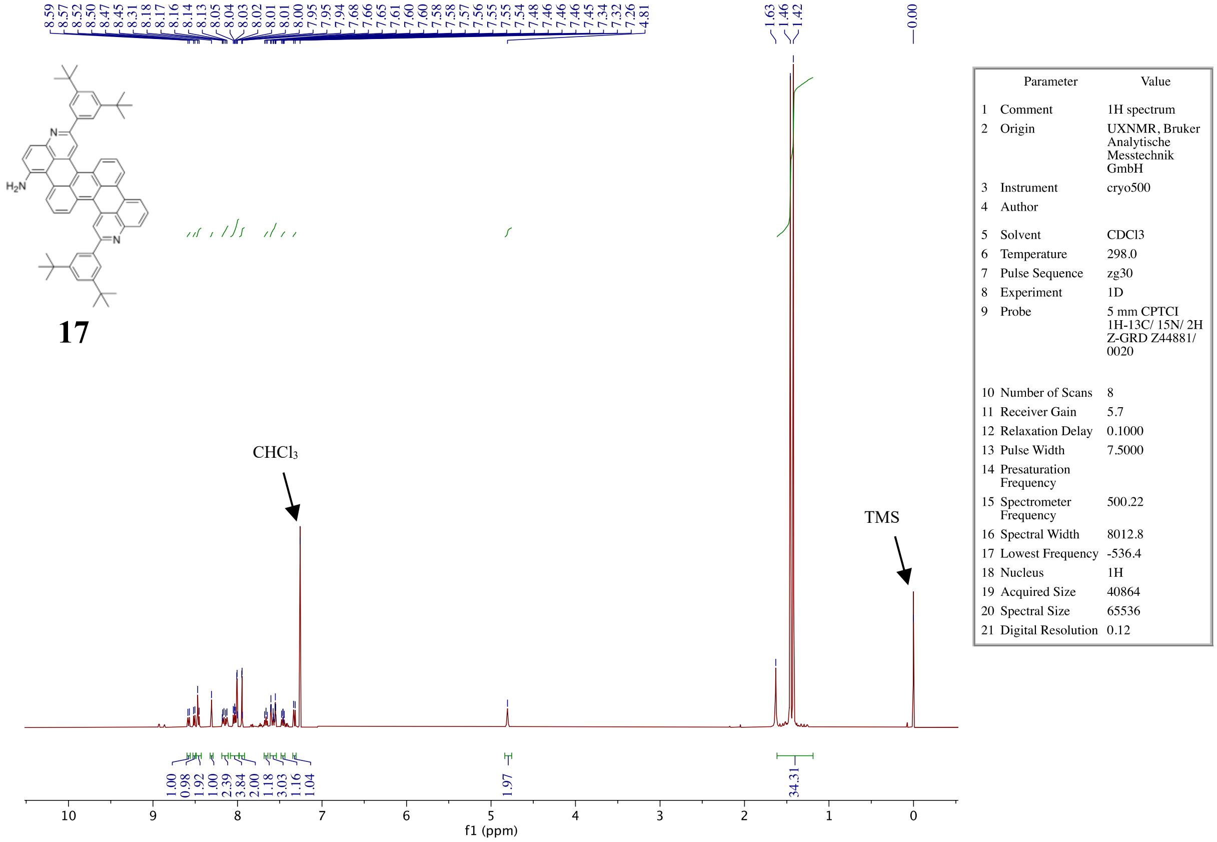

Figure S33. The ^{1}H NMR spectrum obtained for **17**.

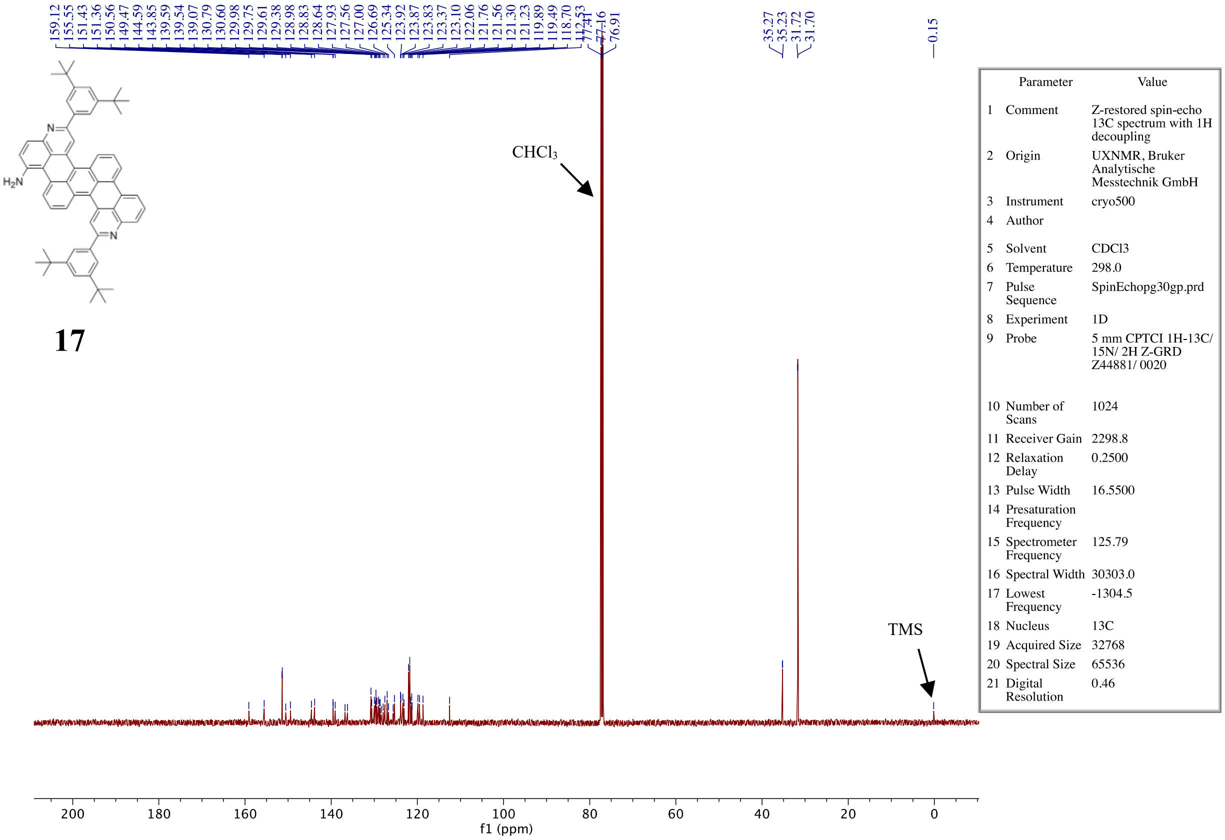

	Parameter	Value
1	Comment	Z-restored spin-echo 13C spectrum with 1H decoupling
2	Origin	UXNMR, Bruker Analytische Messtechnik GmbH
3	Instrument	cryo500
4	Author	
5	Solvent	CDCl3
6	Temperature	298.0
7	Pulse Sequence	SpinEchopg30gp.prd
8	Experiment	1D
9	Probe	5 mm CPTCI 1H-13C/ 15N/ 2H Z-GRD Z44881/ 0020
10	Number of Scans	1024
11	Receiver Gain	2298.8
12	Relaxation Delay	0.2500
13	Pulse Width	16.5500
14	Presaturation Frequency	
15	Spectrometer Frequency	125.79
16	Spectral Width	30303.0
17	Lowest Frequency	-1304.5
18	Nucleus	13C
19	Acquired Size	32768
20	Spectral Size	65536
21	Digital Resolution	0.46

Figure S34. The ^{13}C NMR spectrum obtained for **17**.

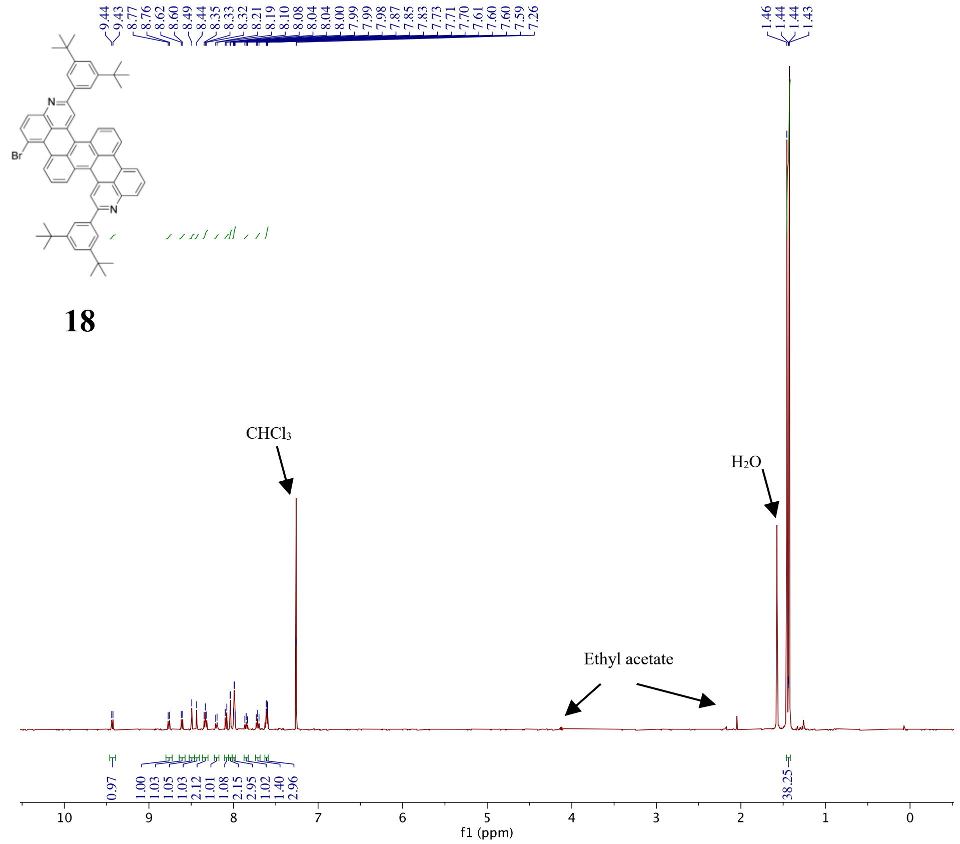

	Parameter	Value
1	Comment	1H spectrum
2	Origin	Bruker Analytik GmbH
3	Instrument	gn500
4	Author	
5	Solvent	CDCl3
6	Temperature	298.0
7	Pulse Sequence	zg30
8	Experiment	1D
9	Probe	5 mm broadband observe Z-grad Z8007/ 19
10	Number of Scans	8
11	Receiver Gain	1448.2
12	Relaxation Delay	0.1000
13	Pulse Width	12.0000
14	Presaturation Frequency	
15	Spectrometer Frequency	499.07
16	Spectral Width	8012.8
17	Lowest Frequency	-544.9
18	Nucleus	1H
19	Acquired Size	40864
20	Spectral Size	65536
21	Digital Resolution	0.12

Figure S35. The ^{1}H NMR spectrum obtained for **18**.

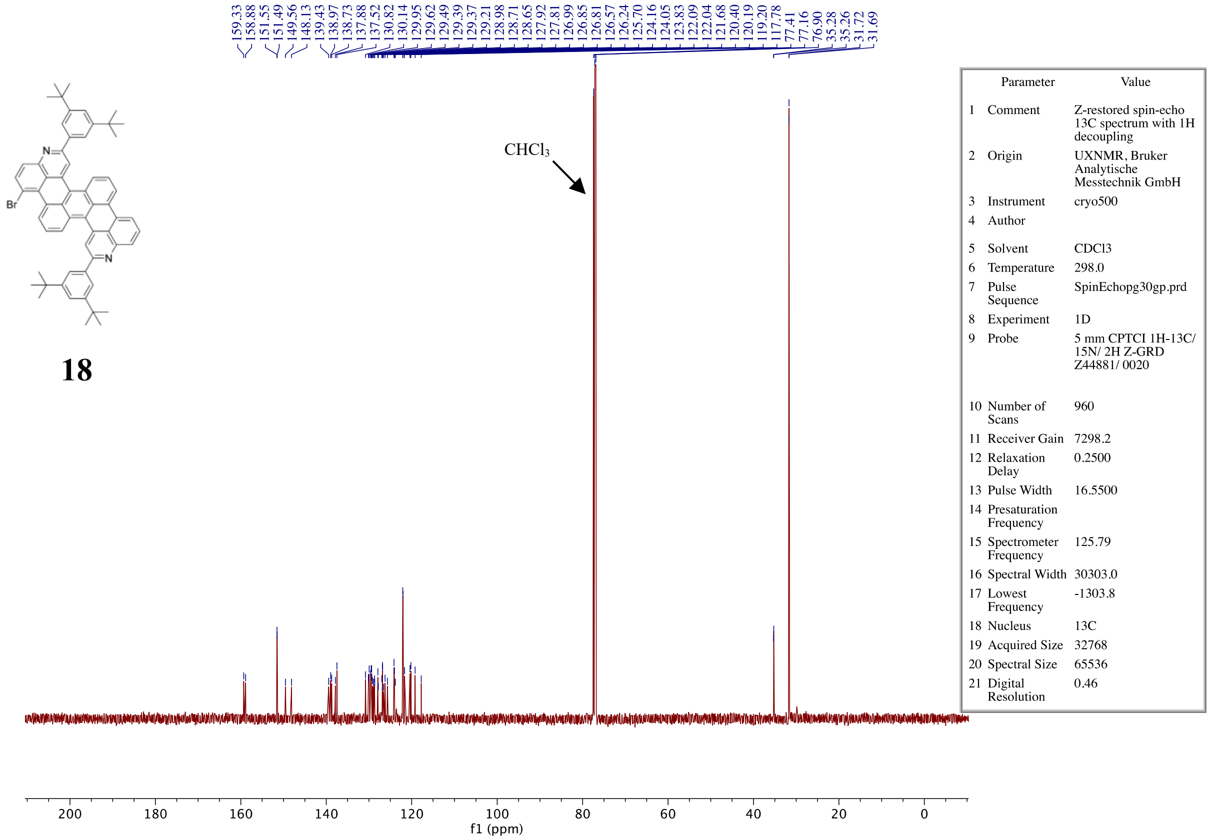

18

	Parameter	Value
1	Comment	Z-restored spin-echo 13C spectrum with 1H decoupling
2	Origin	UXNMR, Bruker Analytische Messtechnik GmbH
3	Instrument	cryo500
4	Author	
5	Solvent	CDCl3
6	Temperature	298.0
7	Pulse Sequence	SpinEchopg30gp.prd
8	Experiment	1D
9	Probe	5 mm CPTCI 1H-13C/ 15N/ 2H Z-GRD Z44881/ 0020
10	Number of Scans	960
11	Receiver Gain	7298.2
12	Relaxation Delay	0.2500
13	Pulse Width	16.5500
14	Presaturation Frequency	
15	Spectrometer Frequency	125.79
16	Spectral Width	30303.0
17	Lowest Frequency	-1303.8
18	Nucleus	13C
19	Acquired Size	32768
20	Spectral Size	65536
21	Digital Resolution	0.46

Figure S36. The ^{13}C NMR spectrum obtained for **18**.

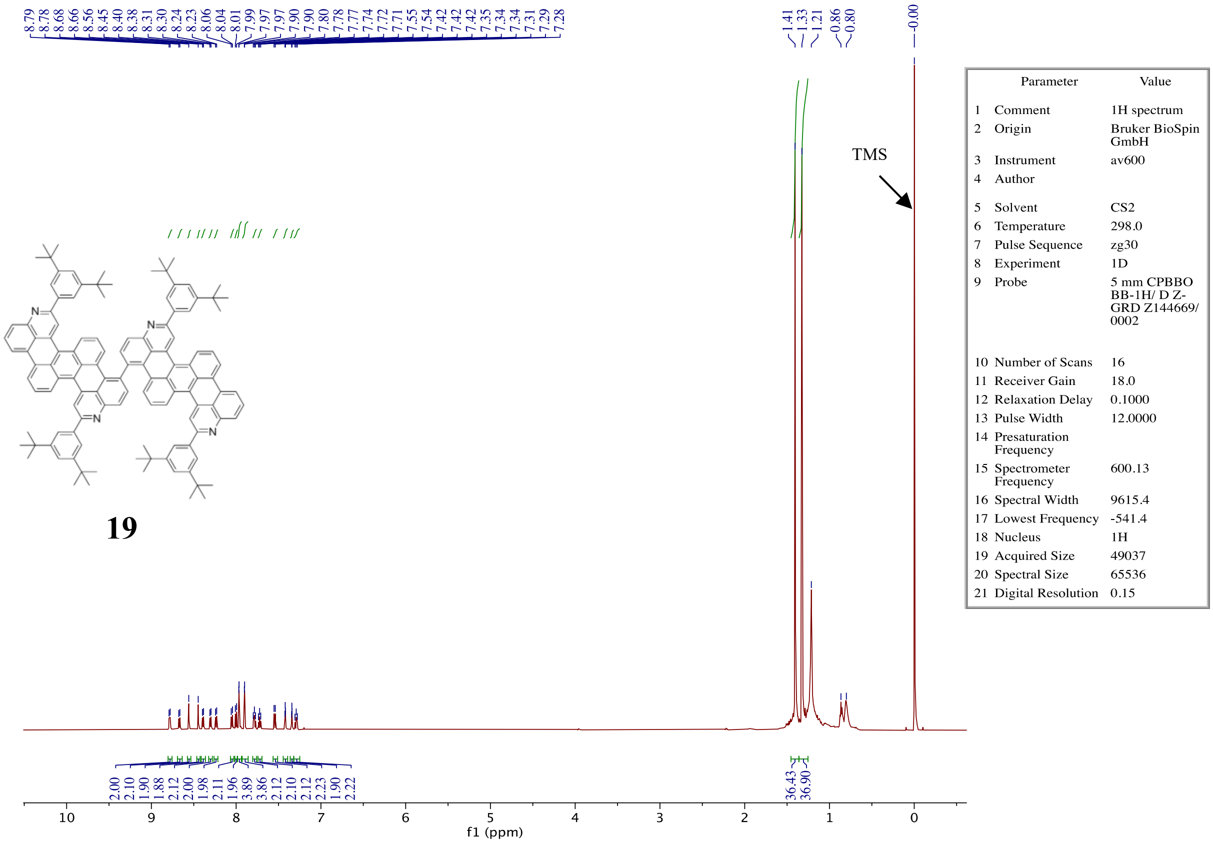

	Parameter	Value
1	Comment	1H spectrum
2	Origin	Bruker BioSpin GmbH
3	Instrument	av600
4	Author	
5	Solvent	CS2
6	Temperature	298.0
7	Pulse Sequence	zg30
8	Experiment	1D
9	Probe	5 mm CPBBO BB-1H/ D Z-GRD Z144669/ 0002
10	Number of Scans	16
11	Receiver Gain	18.0
12	Relaxation Delay	0.1000
13	Pulse Width	12.0000
14	Presaturation Frequency	
15	Spectrometer Frequency	600.13
16	Spectral Width	9615.4
17	Lowest Frequency	-541.4
18	Nucleus	1H
19	Acquired Size	49037
20	Spectral Size	65536
21	Digital Resolution	0.15

Figure S37. The ^{1}H NMR spectrum obtained for **19**.

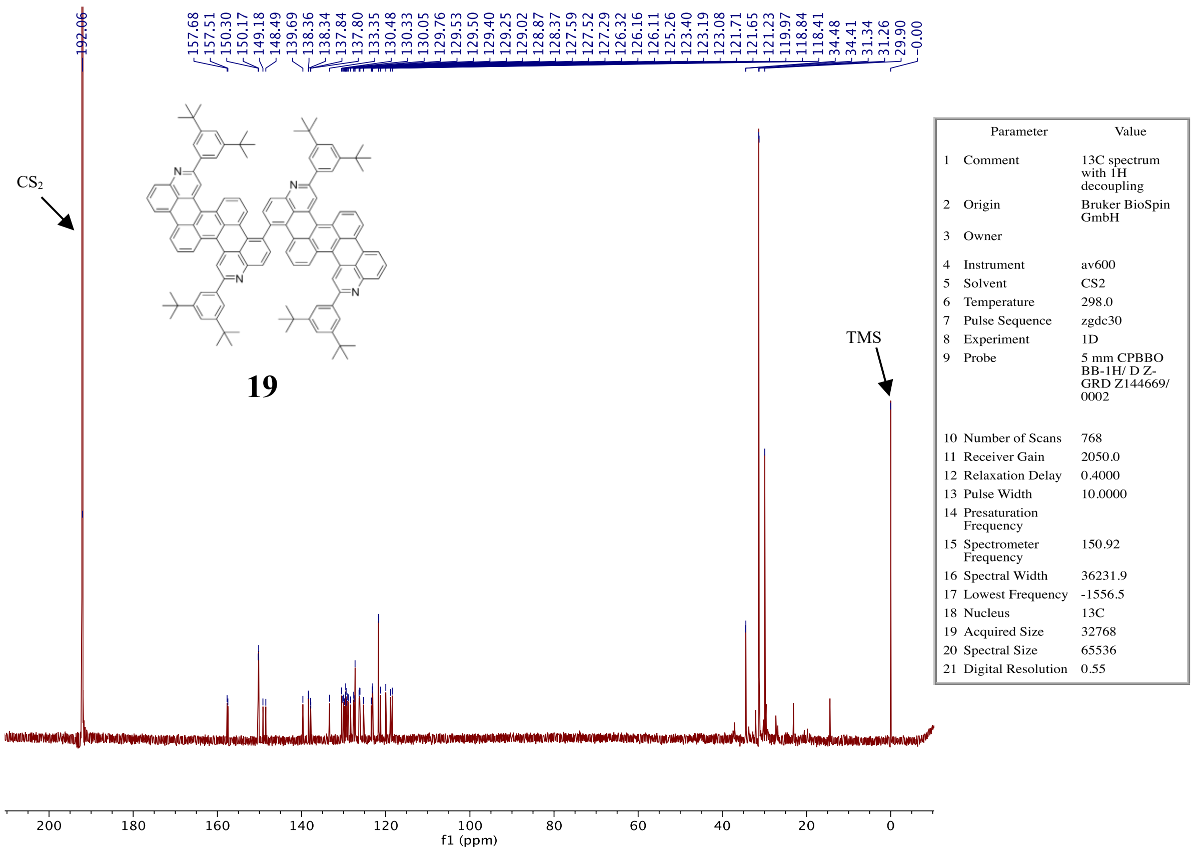

	Parameter	Value
1	Comment	13C spectrum with 1H decoupling
2	Origin	Bruker BioSpin GmbH
3	Owner	
4	Instrument	av600
5	Solvent	CS2
6	Temperature	298.0
7	Pulse Sequence	zgdc30
8	Experiment	1D
9	Probe	5 mm CPBBO BB-1H/ D Z-GRD Z144669/ 0002
10	Number of Scans	768
11	Receiver Gain	2050.0
12	Relaxation Delay	0.4000
13	Pulse Width	10.0000
14	Presaturation Frequency	
15	Spectrometer Frequency	150.92
16	Spectral Width	36231.9
17	Lowest Frequency	-1556.5
18	Nucleus	13C
19	Acquired Size	32768
20	Spectral Size	65536
21	Digital Resolution	0.55

Figure S38. The ^{13}C NMR spectrum obtained for **19**.

II. UV-Vis Spectroscopy Data

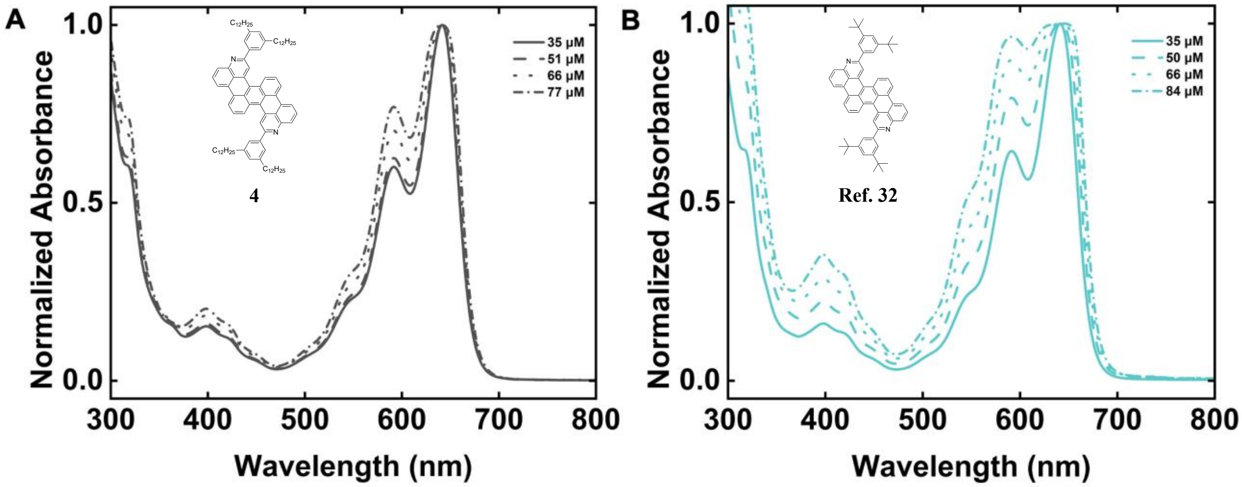

Figure S39. **(A)** The UV-Vis absorption spectra obtained for dodecyl-substituted tetrabenzopentacene **4** at different concentrations. **(B)** The UV-Vis absorption spectra measured for a previously-reported tert-butyl-substituted tetrabenzopentacene at different concentrations (see reference 32). Note that the absorption spectra were normalized by using their maximum absorbance peak within the 600 nm to 700 nm wavelength range in order to facilitate direct comparisons.
